# Impact of Postdiagnosis Health Behaviors and Behavior Changes on Prognosis in Colorectal Cancer Patients: Evidence From Real‐World Data

**DOI:** 10.1002/cam4.71461

**Published:** 2025-12-21

**Authors:** Donghyun Won, Ji Yoon Baek, Ji Won Park, Jeeyoo Lee, Sooyoung Cho, Aesun Shin

**Affiliations:** ^1^ Department of Preventive Medicine Seoul National University College of Medicine Seoul Korea; ^2^ Integrated Major in Innovative Medical Science Seoul National University Graduate School Seoul Korea; ^3^ Department of Surgery Seoul National University Hospital Seoul Korea; ^4^ Cancer Research Institute Seoul National University Seoul Korea; ^5^ Genomic Medicine Institute, Medical Research Center Seoul National University Seoul Korea; ^6^ Interdisciplinary Program in Cancer Biology Major Seoul National University College of Medicine Seoul Korea

**Keywords:** cancer epidemiology, colorectal cancer, long‐term survival, postdiagnosis behaviors

## Abstract

**Background:**

Colorectal cancer (CRC) is a prevalent cancer worldwide, but encouraging healthier lifestyle choices among survivors may improve their prognosis. We aimed to evaluate health habits adopted after diagnosis and their impact on prognosis.

**Methods:**

For this population‐based retrospective cohort study, we used data from the Cancer Public Library Database (CPLD), which consists of four major population‐based public sources in Korea with cancer patients diagnosed between 2012 and 2019. Information on anthropometric measures, physical activity, alcohol consumption, and smoking status before and after cancer diagnosis was used. Hazard ratios (HRs) for all‐cause deaths with 95% confidence intervals (CIs) were estimated via the Cox proportional hazards model.

**Results:**

Among the 7553 CRC patients, postdiagnosis physical activity was significantly related to decreased risk of death among those who were diagnosed with stage I or III CRC (*p* for trend < 0.05). The analysis of 4588 patients revealed that increased physical activity (stage I: adjusted HR [aHR] = 0.60, 95% CI = 0.29–1.22; stage II: aHR = 0.86, 95% CI = 0.49–1.52; stage III: aHR = 0.60, 95% CI = 0.36–0.99) or smoking cessation (stage I: aHR = 0.81, 95% CI = 0.28–2.35; stage II: aHR = 0.36, 95% CI = 0.16–0.81; stage III: aHR = 0.89, 95% CI = 0.42–1.87), were associated with poor prognosis compared to those who consistently remained physically inactive or continued to smoke.

**Conclusion:**

The present study provides compelling evidence on the benefits of increased physical activity and smoking cessation after CRC diagnosis for improved survival.

## 1 Introduction

Over 1.9 million new cases of colorectal cancer (CRC) and 900,000 CRC‐related deaths were estimated in 2022 [1]. CRC is the third most commonly diagnosed cancer, and the second leading cause of cancer‐related mortality worldwide [2]. Meanwhile, 5‐year survival rates of CRC have improved, ranging from 50% to over 70% in most developed countries, which shows regional discrepancies [2, 3].

A cancer survivor is defined as a person who has had a cancer diagnosis, including people who had no signs of cancer after finishing treatment and who experienced either recurrence, metastasis, or secondary cancer [4]. In particular, the risk of recurrence, secondary malignancies, or comorbidities has been reported to be greater in CRC survivors, which highlights the importance of investigating survival to improve the prognosis of this disease [5, 6, 7].

Lifestyle factors such as obesity, physical inactivity, regular alcohol consumption, cigarette smoking, and high consumption of red or processed meats are known risk factors for CRC [8]. The World Cancer Research Fund (WCRF) and the American Institute for Cancer Research (AICR) recommended avoiding these risk factors, anticipating a better prognosis for this cancer, as well as cancer prevention [9]. However, a previous meta‐analysis of observational studies revealed that the current evidence concerning the effects of postdiagnosis behaviors on improving or worsening CRC prognosis is inconsistent, which emphasizes the need for further research [10].

The Nurses' Health Study showed that significant behavioral changes occur following a cancer diagnosis, as CRC patients often experience psychological distress—such as anxiety or depression. Those with higher levels of psychological distress were more likely to engage in unhealthy habits, highlighting the importance of follow‐up assessments of behaviors after a cancer diagnosis [11]. In addition, cancer stage at diagnosis is the most important prognostic factors [12], therefore, stratifying analysis rather than adjustment of the stage would be beneficial to clarify the potential impact of health behaviors after cancer diagnosis.

Therefore, the current study aimed to assess the impact of health behaviors both before and after CRC diagnosis on survival, with stratification by cancer stage in real‐world patient data.

## 2 Materials and Methods

### 2.1 Data Source

The Cancer Public Library Database is a nationwide longitudinal dataset that ensures reliable representation of registered cancer survivors in Korea. It was established under the Korean Clinical Utilization for Research Excellence (K‐CURE) project and consists of four major population‐based public sources in Korea, described as follows: the Korea National Cancer Incidence Database in the Korea Central Cancer Registry (2012–2019), cause‐of‐death data in Statistics Korea (2012–2020), the National Health Information Database (NHID) in the National Health Insurance Service (2012–2021), and the National Health Insurance Research Database in the Health Insurance Review and Assessment Service (2012–2021) [13].

We used health check‐up examination data from NHID. Behavioral variables were based on self‐reported responses, while anthropometric measurements such as height, weight, and waist circumference were obtained by trained medical staff. For participants who received multiple health check‐up examinations, we selected the data closest to the date of cancer diagnosis. We also utilized an eligibility database from the NHID to determine the socioeconomic status of the study population. The linkage of cause‐of‐death data in Statistics Korea was further used to obtain details about the date of death among the study population.

Individuals with International Classification of Diseases for Oncology codes, 3rd edition of C18–C20, were registered in the Korea Central Cancer Registry as patients with CRC. We used a CRC database provided by the K‐CURE that included a randomly selected 10% sample of the total number of registered CRC survivors in Korea between 2012 and 2019 (*n* = 21,741).

### 2.2 Patient Selection (Study Population)

**FIGURE 1:**
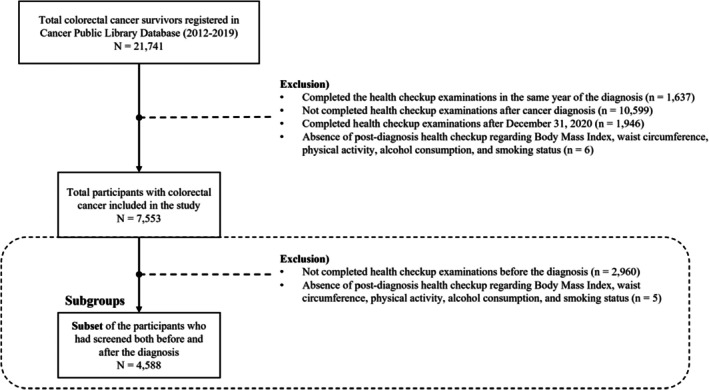
Flow chart of study population from Cancer Public Library Database (2012–2019).

Among 21,741 patients registered in the CRC database, we excluded participants who completed health check‐up examinations in the same year as their diagnosis (*n* = 1637) and those who did not complete them after their cancer diagnosis (*n* = 10,599) to clarify that the time of diagnosis preceded the health check‐up examinations. We then excluded participants who completed the health check‐up examinations after December 31, 2020 (*n* = 1946), when the last date the cause of death data was available. A total of 7553 participants were included in the study after finally excluding participants who were missing in main postdiagnosis behavior variables (*n* = 6). A subgroup of 4588 participants who participated in health check‐ups both before and after cancer diagnosis were analyzed to address the impact of behavioral changes on prognosis (Figure 1).

### 2.3 Postdiagnosis Health Behaviors

We employed the standardized scoring system of the 2018 WCRF/AICR cancer prevention recommendations to evaluate behaviors, classifying participants into “poor,” “moderate,” and “good” groups according to the criteria [9, 14]. The criteria were adapted according to the context of Korea [15]. For BMI and waist circumference, which are available for each unit of 1 kg/m^2^ and 10 cm from the database, we adapted the scoring system referred to the Korean Society for the Study of Obesity [16]. The criterion for physical activity referred to that of the World Health Organization, where patients were classified into three groups based on the reported frequency of moderate to vigorous physical activity per week: 0 days, 1–4 days or more than 5 days [17]. Third, patients were categorized as heavy drinkers (men: ≥ 28 g of ethanol/day or 2 drinks/day; women: ≥ 14 g of ethanol/day or 1 drink/day), light drinkers (men: 0–< 28 g of ethanol/day or 0–< 2 drinks/day; women: 0–< 14 g of ethanol/day or 0–< 1 drink/day), or nondrinkers, following the criteria of WCRF/AICR cancer prevention recommendations. Since the information on alcohol consumption by beverage type was not collected prior to 2018, total intake was calculated based on drinking frequency and quantity; from 2018 onward, we’ve calculated beverage‐specific amount of ethanol, where the reference volume of commonly consumed alcoholic drinks in Korea was determined according to the low‐risk drink guidelines: 50 mL per shot glass of soju, 250 mL per glass of beer, 35 mL per shot glass of liquor, 150 mL per shot glass of Korean liquor, and 125 mL per glass of wine [18]. For those who responded only to the frequency of consumption without specifying the amount, the volume was estimated using the median consumption amount by sex. Smoking status was classified into three groups: current smokers, former smokers, or nonsmokers.

### 2.4 Covariates

Age, sex, and cancer stage data were collected at the time of cancer diagnosis from the Korea National Cancer Incidence Database. We considered cancer stages I–IV or unknown, which were derived from the 7th edition of the American Joint Committee on Cancer (AJCC) [19]. Insurance type, income level, and residential area were categorized by status in December of the year of the cancer diagnosis. We classified insurance types as local insured, employee insured, medical‐aid beneficiary or missing and divided into tertiles according to insurance premium. Residential areas were grouped into three categories: Seoul; Metropolitan cities, including Busan, Daegu, Incheon, Gwangju, Daejeon, Ulsan, and Sejong; and other regions, including Gyeonggi‐do, Gangwon‐do, Chungcheongbuk‐do, Chungcheongnam‐do, Jeollabuk‐do, Jeollanam‐do, Gyeongsangbuk‐do, Gyeongsangnam‐do, and Jeju. Disease history was collected from the questionnaire postdiagnosis health check‐up examinations. We defined patients with hypertension as those whose systolic blood pressure was ≥ 140 mmHg, whose diastolic blood pressure was ≥ 90 mmHg, or whose history of hypertension was reported. Patients with diabetes were also defined as those whose fasting blood glucose was ≥ 126 mg/dL or those who reported a history of diabetes.

### 2.5 Statistical Analysis

The distribution of general characteristics was summarized by the number and percentages of the participants for each variable. A Bowker's matched‐pairs test was conducted to examine changes in adherence patterns following the cancer diagnosis. The associations between postdiagnosis behaviors and all‐cause death were assessed using a Cox proportional hazard model, with hazard ratios (HRs) and corresponding 95% confidence intervals (CIs). We adjusted for patient age, sex, year postdiagnosis, cancer stage, insurance type, income level, and residence according to previous studies [20, 21]. The potential confounding impact of each covariate was also evaluated through chi‐square tests and univariate survival analyses among the study participants. For covariates with missing data (e.g., income level), missing values were categorized as a distinct group and incorporated into the model. For each participant, the follow‐up period started at the date of the postdiagnosis health check‐up examinations, considering the immortal time. Since health check‐up dates were only available by year, January was assumed as the month of examination to align with monthly data on cancer diagnosis and death. The follow‐up period ended with the date of death or December 31, 2020. Subgroup analyses were conducted according to cancer stage, age group, and cancer sites, with the latter two presented in the Tables S1–S4. Considering the distinct clinical characteristics of patients diagnosed with CRC at stage IV and its small sample size, stage I–III cases were included for detailed subgroup analysis. A two‐sided *p* value of < 0.05 was considered statistically significant for all the statistical analyses. The assessment of the associations between pre‐ and postdiagnosis behavioral changes and all‐cause death was conducted using the methods described above. A sensitivity analysis was conducted using the same approach, with the follow‐up period initiated at the date of cancer diagnosis, described in Table S5.

All the data analysis was conducted utilizing SAS statistical software package version 9.4 (SAS Institute Inc., Cary, NC, USA) [22]. The study was approved by the Institutional Review Boards of Seoul National University Hospital (IRB no. E‐2312‐132‐1496), and the procedures followed were in accordance with the ethical standards of the Helsinki Declaration.

## 3 Results

### 3.1 Demographic and Clinical Differences in CRC by Cancer Stage

Table 1 shows the characteristics of CRC survivors by cancer stage. Among the 7553 CRC patients, 4614 were men and 2939 were women. More than 80% of the study population was aged 50–79 years, and the percentage of the employee insured population was 68.1%. With respect to cancer stage, 2685 (35.5%), 2087 (27.5%), and 2250 (29.8%) patients were diagnosed with stage I, II, and III CRC, respectively. Compared to them, patients diagnosed with CRC at stage IV were found to be younger at diagnosis and have lower income levels (38.1%). Additionally, the proportion of those with a history of hypertension was lower (40.9%), while that of diabetes was higher (28.1%).

**TABLE 1:** Basic characteristics among colorectal cancer survivors from Cancer Public Library Database by cancer stage at diagnosis (2012–2019).

Cancer stage (AJCC‐7)	Study population (*N* = 7553)				
	I (*N* = 2685)	II (*N* = 2087)	III (*N* = 2250)	IV (*N* = 367)	Unknown (*N* = 164)
Age at diagnosis					
< 40	57 (2.1)	49 (2.3)	65 (2.9)	11 (3.0)	7 (4.3)
40–49	185 (6.9)	171 (8.2)	223 (9.9)	39 (10.6)	11 (6.7)
50–59	704 (26.2)	489 (23.4)	641 (28.5)	120 (32.7)	39 (23.8)
60–69	936 (34.9)	619 (29.7)	694 (30.8)	114 (31.1)	55 (33.5)
70–79	704 (26.2)	605 (29)	520 (23.1)	75 (20.4)	42 (25.6)
≥ 80	99 (3.7)	154 (7.4)	107 (4.8)	8 (2.2)	10 (6.1)
Sex					
Men	1669 (62.2)	1257 (60.2)	1349 (60.0)	234 (63.8)	105 (64.0)
Women	1016 (37.8)	830 (39.8)	901 (40.0)	133 (36.2)	59 (36.0)
Years since diagnosis (Mean, SD)	0.8, 0.5	0.8, 0.5	0.8, 0.5	0.7, 0.5	0.8, 0.5
Insurance type					
Local insured	777 (28.9)	638 (30.6)	648 (28.8)	110 (30.0)	49 (29.9)
Employee insured	1842 (68.6)	1392 (66.7)	1552 (69.0)	251 (68.4)	110 (67.1)
Medical‐aid beneficiary	66 (2.5)	57 (2.7)	50 (2.2)	6 (1.6)	5 (3.0)
Income level (decile)					
Lowest (0%–30%)	873 (32.5)	691 (33.1)	777 (34.5)	140 (38.1)	54 (32.9)
Middle (40%–70%)	712 (26.5)	542 (26.0)	574 (25.5)	100 (27.2)	54 (32.9)
Highest (80%–100%)	1047 (39.0)	812 (38.9)	847 (37.6)	118 (32.2)	53 (32.3)
Missing	53 (2.0)	42 (2.0)	52 (2.3)	9 (2.5)	3 (1.8)
Residence^a^					
Seoul	432 (16.1)	309 (14.8)	353 (15.7)	77 (21.0)	20 (12.2)
Metropolitan cities	804 (29.9)	626 (30.0)	686 (30.5)	103 (28.1)	54 (32.9)
Others	1449 (54.0)	1152 (55.2)	1211 (53.8)	187 (51.0)	90 (54.9)
History of hypertension					
No	1293 (48.2)	1061 (50.8)	1258 (55.9)	217 (59.1)	79 (48.2)
Yes^b^	1383 (51.5)	1023 (49.0)	988 (43.9)	150 (40.9)	85 (51.8)
Missing	9 (0.3)	3 (0.1)	4 (0.2)	0 (0.0)	0 (0.0)
History of diabetes					
No	1983 (73.9)	1528 (73.2)	1660 (73.8)	259 (70.6)	111 (67.7)
Yes^c^	667 (24.8)	527 (25.3)	563 (25.0)	103 (28.1)	52 (31.7)
Missing	35 (1.3)	32 (1.5)	27 (1.2)	5 (1.4)	1 (0.6)

Abbreviations: AJCC, American Joint Committee on Cancer; *N*, the number of corresponding cases.

^a^ Metropolitan cities include Busan, Daegu, Incheon, Gwangju, Daejeon, Ulsan, Sejong in Korea. Other regions include Gyeonggi, Gangwon, Chungbuk, Chungnam, Jeonbuk, Jeonnam, Gyeongbuk, Gyeongnam, Jeju in Korea.

^b^ Defined as those who were measured as having ≥ 140 of systolic blood pressure or ≥ 90 of diastolic blood pressure, or those who self‐reported hypertension.

^c^ Defined as those who were measured as having ≥ 126 of fasting blood glucose, or those who self‐reported the history of diabetes.

### 3.2 Patterns of Behavioral Changes Surrounding Cancer Diagnosis

Table 2 shows trends of behavior changes among 4588 CRC patients whose health check‐up examinations both before and after their cancer diagnosis were available. Statistically significant differences were observed across all four behaviors. Among those participants, 22.0% (*n* = 1011) stably maintained a healthy weight, defined as a BMI between 19.0 and 23.0 and waist circumference below 90 cm for men 80 cm for women. Another 33.1% (*n* = 1537) experienced shifts in weight status in both directions—either from healthy to unhealthy or vice versa, based on the defined criteria. Among 45.9% (*n* = 2106) of the patients who reported changes in pre‐ and postdiagnosis behaviors, 63.0% (*n* = 1326) of them reported increased physical activity. With respect to alcohol consumption and smoking habits, more than 85% of the participants decreased their consumption after their cancer diagnosis or remained in a lower consumption group.

**TABLE 2:** Behavior changes between pre‐ and post‐colorectal cancer diagnosis.

Prediagnosis behaviors	Postdiagnosis behaviors (*N* = 4588)			*p* ^a^
	*N* (%)	*N* (%)	*N* (%)	
Be a healthy weight^b^				
Poor	1227 (70.4)	346 (24.3)	104 (7.3)	0.001
Moderate	431 (24.7)	813 (57.2)	309 (21.7)	
Good	84 (4.9)	263 (18.5)	1011 (71.0)	
Be physically active^c^				
Poor	1231 (66.3)	1075 (44.0)	112 (38.6)	< 0.001
Moderate	545 (29.4)	1212 (49.6)	139 (47.9)	
Good	80 (4.3)	155 (6.4)	39 (13.5)	
Limit alcohol consumption^d^				
Poor	285 (75.6)	239 (28.1)	487 (14.5)	< 0.001
Moderate	67 (17.8)	383 (45.1)	614 (18.3)	
Good	25 (6.6)	228 (26.8)	2260 (67.2)	
Quit smoking^e^				
Poor	203 (89.8)	572 (36.2)	171 (6.1)	< 0.001
Moderate	14 (6.2)	776 (49.1)	313 (11.2)	
Good	9 (4.0)	231 (14.7)	2299 (82.7)	

^a^ *p*‐value is for Bowker's matched‐pair test by R version 4.3.1.

^b^ Participants categorized based on BMI (kg/m^2^) and waist circumference (cm)—poor: BMI < 19.0 or BMI ≥ 23.0 with waist circumference ≥ 90 (men) or ≥ 80 (women); moderate: BMI < 19.0 or BMI ≥ 23.0 with waist circumference < 90 (men) or < 80 (women); good: BMI between 19.0 and 23.0 with waist circumference < 90 (men) or < 80 (women).

^c^ Participants categorized based on the number of days of moderate to vigorous physical activity per week—poor: 0 days; moderate: 1–4 days; good group: ≥ 5 days.

^d^ Participants categorized based on the total alcohol consumption—poor: heavy drinkers (men: ≥ 2 drink/day, women: ≥ 1 drink/day); moderate: light drinkers (men: < 2 drink/day, women: < 1 drink/day); good: nondrinkers.

^e^ Participants categorized based on by the smoking status—poor: current smokers; moderate: former smokers; good: nonsmokers.

### 3.3 Postdiagnosis Physical Activity and Body Composition in Relation to Prognosis, by Cancer Stage

Table 3 shows the associations between postdiagnosis behaviors and all‐cause death stratified by cancer stage among 4295 CRC survivors. The mean follow‐up was 0.6 years from the postdiagnosis behavior measurement and 1.4 years from the cancer diagnosis. Postdiagnosis moderate to vigorous physical activity was significantly related to decreased risk of death among those who were diagnosed with stage I (moderate group: adjusted hazard ratio [aHR] = 0.55, 95% confidence interval [95% CI] = 0.37–0.82) or III CRC (moderate group: aHR = 0.63, 95% CI = 0.47–0.82), compared to the poor group who reported no postdiagnosis physical activity. Higher risk of death was shown from the good group whose BMI was between 19.0 and 23.0 with waist circumference below 90 cm for men or 80 cm for women, especially those who were diagnosed with stage II or III CRC (stage II: aHR = 1.43, 95% CI = 1.00–2.04; stage III: aHR = 1.75, 95% CI = 1.30–2.36). Subgroup analyses by cancer site showed consistent results as observed in the overall population, although the associations between postdiagnosis behavior and prognosis were not shown in subgroup analyses stratified by age group (Tables S1 and S2).

**TABLE 3:** Cox proportional hazard models for the associations between postdiagnosis health behaviors and the risk of death by cancer stage.

Group^a^	Stage I (*N* = 2685)			Stage II (*N* = 2087)			Stage III (*N* = 2250)		
	Poor	Moderate	Good	Poor	Moderate	Good	Poor	Moderate	Good
Be a healthy weight^b^									
*N*	1035	871	779	785	648	654	842	681	727
Person‐years	638.3	563.0	493.3	485.2	409.8	421.2	532.7	405.0	425.3
No. of deaths	43	37	47	55	39	80	80	73	112
HR (95% CI)	Ref.	0.97 (0.62–1.50)	1.39 (0.92–2.10)	Ref.	0.83 (0.55–1.26)	**1.67** **(1.19–2.36)**	Ref.	1.20 (0.88–1.65)	**1.76** **(1.32–2.34)**
aHR (95% CI)	Ref.	1.00 (0.63–1.57)	1.33 (0.87–2.04)	Ref.	0.80 (0.53–1.22)	**1.43** **(1.00–2.04)**	Ref.	1.42 (1.03–1.97)	**1.75** **(1.30–2.36)**
Be physically active^c^									
*N*	1117	1404	164	922	1028	137	918	1173	159
Person‐years	787.7	794.8	112.2	632.4	588.4	95.3	601.4	652.7	108.8
No. of deaths	82	37	8	106	55	13	161	83	21
HR (95% CI)	Ref.	**0.45** **(0.30–0.66)**	0.69 (0.33–1.42)	Ref.	**0.54** **(0.39–0.75)**	0.80 (0.45–1.42)	Ref.	**0.48** **(0.37–0.62)**	0.73 (0.46–1.15)
aHR (95% CI)	Ref.	**0.55** **(0.37–0.82)**	0.61 (0.29–1.27)	Ref.	0.76 (0.54–1.06)	1.13 (0.62–2.03)	Ref.	**0.63** **(0.47–0.82)**	0.97 (0.61–1.55)
Limit alcohol consumption^d^									
*N*	311	624	1750	170	366	1551	140	344	1766
Person‐years	188.2	330.8	1175.7	94.5	192.7	1029.0	61.3	173.3	1128.3
No. of deaths	11	16	100	12	16	146	11	16	238
HR (95% CI)	Ref.	0.85 (0.39–1.82)	1.46 (0.78–2.72)	Ref.	0.65 (0.31–1.37)	1.11 (0.62–2.00)	Ref.	0.51 (0.24–1.10)	1.17 (0.64–2.14)
aHR (95% CI)	Ref.	0.76 (0.35–1.65)	1.18 (0.62–2.27)	Ref.	0.71 (0.33–1.51)	1.02 (0.55–1.89)	Ref.	0.47 (0.22–1.02)	0.85 (0.46–1.57)
Quit smoking^e^									
*N*	193	909	1583	118	679	1290	116	752	1382
Person‐years	176.5	537.2	980.9	106.0	396.4	813.8	93.8	439.2	829.9
No. of deaths	19	34	74	15	60	99	22	74	169
HR (95% CI)	Ref.	0.61 (0.35–1.07)	0.72 (0.43–1.19)	Ref.	1.10 (0.62–1.94)	0.89 (0.52–1.53)	Ref.	0.72 (0.45–1.16)	0.87 (0.56–1.36)
aHR (95% CI)	Ref.	0.58 (0.33–1.02)	0.73 (0.42–1.28)	Ref.	0.90 (0.51–1.59)	0.73 (0.41–1.31)	Ref.	0.63 (0.39–1.02)	0.72 (0.45–1.56)

*Note:* Bold values denote statistically significant results.

Abbreviations: aHR, adjusted hazard ratios; BMI, body mass index; CI, confidence interval; N/No., the number of corresponding cases; Ref., reference.

^a^ The models were adjusted for age, sex (men or women), year postdiagnosis, insurance type (local insured, employee insured or medical‐aid beneficiary), income level (decile to tertile; tertile 1 (0, 1, 2, 3, 4), tertile 2 (5, 6, 7), tertile 3 (8, 9, 10) or missing), and residence (Seoul, metropolitan cities or others).

^b^ Participants categorized based on BMI (kg/m^2^) and waist circumference (cm)—poor: BMI < 19.0 or BMI ≥ 23.0 with waist circumference ≥ 90 (men) or ≥ 80 (women); moderate: BMI < 19.0 or BMI ≥ 23.0 with waist circumference < 90 (men) or < 80 (women); good: BMI between 19.0 and 23.0 with waist circumference < 90 (men) or < 80 (women).

^c^ Participants categorized based on the number of days of moderate to vigorous physical activity per week—poor: 0 days; moderate: 1–4 days; good group: ≥ 5 days.

^d^ Participants categorized based on the total alcohol consumption—poor: heavy drinkers (men: ≥ 2 drink/day, women: ≥ 1 drink/day); moderate: light drinkers (men: < 2 drink/day, women: < 1 drink/day); good: nondrinkers.

^e^ Participants categorized based on smoking status—poor: current smokers; moderate: former smokers; good: nonsmokers.

### 3.4 Stage‐Specific Benefits of Behavioral Improvements—Especially Regarding the Physical Activity and Smoking

Table 4 shows the associations between behavior changes and all‐cause death by cancer stage. The average follow‐up period was 0.5 years from the postdiagnosis behavior assessment and 1.2 years from the time of cancer diagnosis. Improving or maintaining behaviors as recommended was related to a lower risk of all‐cause death, specifically for behaviors such as physical activity or smoking. Notably, among individuals diagnosed at stage I, those in the stable good group showed a significantly reduced risk of death (aHR = 0.35, 95% CI = 0.13–0.94), while at stage III, the enhanced group demonstrated a similar protective association (aHR = 0.60, 95% CI = 0.36–0.99). In terms of smoking behavior, all groups—worsen, enhanced, and stable good—showed lower risk of death compared to the reference group, except for the worsen group in stage I. Specifically, statistically significant results were observed among patients diagnosed CRC at stage II (enhanced: aHR = 0.36, 95% CI = 0.16–0.81; stable good: aHR = 0.27, 95% CI = 0.13–0.55). These associations were also shown in the subgroup analyses by age and cancer sites, except among early onset CRC patients due to their small sample size (Tables S3 and S4). A sensitivity analysis that started follow‐up at the date of cancer diagnosis revealed an association between behavior changes and survival, as shown in Table S5.

**TABLE 4:** Cox proportional hazard models for the association between behavior changes and the risk of death by cancer stage.

Behavior changes^a^	Stage I (*n* = 1716)				Stage II (*n* = 1246)				Stage III (*n* = 1333)			
	Unchanged (poor)^b^	Worsen^c^	Enhanced^d^	Stable (good)^e^	Unchanged (poor)^b^	Worsen^c^	Enhanced^d^	Stable (good)^e^	Unchanged (poor)^b^	Worsen^c^	Enhanced^d^	Stable (good)^e^
Be a healthy weight^f^												
*N*	468	292	264	692	350	191	200	505	334	240	236	523
Person‐years	258.9	137.1	132.1	368.9	192.3	94.3	122.5	268.2	172.9	117.6	114.8	265.3
No. of deaths	12	9	8	26	25	5	17	34	27	15	28	54
HR (95% CI)	Ref.	1.47 (0.62–3.48)	1.30 (0.53–3.19)	1.52 (0.77–3.02)	Ref.	0.41 (0.16–1.08)	1.09 (0.59–2.01)	0.98 (0.59–1.65)	Ref.	0.82 (0.44–1.54)	1.56 (0.92–2.64)	1.31 (0.83–2.09)
aHR (95% CI)	Ref.	1.67 (0.93–2.00)	1.72 (0.69–4.27)	1.87 (0.93–3.76)	Ref.	0.51 (0.19–1.33)	1.18 (0.63–2.21)	1.11 (0.66–1.87)	Ref.	1.06 (0.56–2.01)	**1.75** **(1.03–2.99)**	1.55 (0.98–2.48)
Be physically active^g^												
*N*	457	288	487	484	374	205	339	328	332	222	408	371
Person‐years	26	13	11	5	381	14	19	10	49	32	23	20
No. of deaths	281.0	162.1	225.1	228.8	225.1	113.2	174.9	164.1	182.6	117.1	192.8	178.2
HR (95% CI)	Ref.	0.84 (0.43–1.64)	0.54 (0.26–1.09)	**0.24** **(0.90–0.61)**	Ref.	0.73 (0.39–1.34)	0.64 (0.37–1.10)	**0.36** **(0.18–0.72)**	Ref.	1.10 (0.65–1.58)	**0.44** **(0.27–0.73)**	**0.42** **(0.25–0.70)**
aHR (95% CI)	Ref.	0.86 (0.44–1.71)	0.60 (0.29–1.22)	**0.35** **(0.13–0.94)**	Ref.	0.86 (0.46–1.60)	0.86 (0.49–1.52)	0.52 (0.25–1.07)	Ref.	1.31 (0.83–2.08)	**0.60** **(0.36–0.99)**	0.68 (0.40–1.18)
Limit alcohol consumption^h^												
*N*	140	155	421	1000	75	78	345	748	56	74	440	763
Person‐years	3	3	13	36	6	3	23	49	1	4	40	79
No. of deaths	60.5	62.8	237.4	536.3	35.6	36.2	196.0	409.5	16.7	26.3	252.7	375.1
HR (95% CI)	Ref.	0.98 (0.20–4.87)	1.09 (0.33–3.84)	1.33 (0.41–4.31)	Ref.	0.49 (0.12–1.94)	0.69 (0.28–1.71)	0.71 (0.30–1.65)	Ref.	2.49 (0.28–22.27)	2.64 (0.36–19.26)	3.51 (0.49–25.29)
aHR (95% CI)	Ref.	0.72 (0.14–3.62)	0.91 (0.26–3.25)	0.80 (0.24–2.67)	Ref.	0.55 (0.14–2.23)	0.68 (0.27–1.68)	0.54 (0.23–1.27)	Ref.	1.68 (0.19–15.27)	1.89 (0.26–13.89)	1.76 (0.24–12.84)
Quit smoking^i^												
*N*	80	109	394	1133	47	57	282	860	57	73	305	898
Person‐years	61.8	55.3	187.7	592.3	33.4	28.8	131.3	483.7	36.7	43.8	146.2	444.0
No. of deaths	5	6	12	32	10	3	15	53	9	4	32	79
HR (95% CI)	Ref.	1.40 (0.43–4.59)	0.84 (0.29–2.39)	0.69 (0.27–1.78)	Ref.	0.34 (0.09–1.25)	**0.39** **(0.17–0.86)**	**0.37** **(0.19–0.73)**	Ref.	0.37 (0.11–1.20)	0.88 (0.42–1.85)	0.72 (0.36–1.44)
aHR (95% CI)	Ref.	1.16 (0.35–3.89)	0.81 (0.28–2.35)	0.59 (0.22–1.55)	Ref.	0.29 (0.01–1.07)	**0.36** **(0.16–0.81)**	**0.27** **(0.13–0.55)**	Ref.	**0.24** **(0.07–0.77)**	0.89 (0.42–1.87)	0.54 (0.27–1.09)

*Note:* Bold values denote statistically significant results.

Abbreviations: aHR, adjusted hazard ratios; BMI, body mass index; CI, confidence interval; N/No., the number of corresponding cases; Ref., reference.

^a^ The models were adjusted for age, sex (men or women), year postdiagnosis, insurance type (local insured, employee insured or medical‐aid beneficiary), income level (decile to tertile; tertile 1 (0, 1, 2, 3, 4), tertile 2 (5, 6, 7), tertile 3 (8, 9, 10) or missing), and residence (Seoul, metropolitan cities or others).

^b^ “Unchanged (poor)” refers to individuals whose health behaviors remained poor from pre‐ to post‐assessment (i.e., poor–poor).

^c^ “Worsen” refers to a worsen in health behaviors, including transitions from moderate to poor, good to poor, or good to moderate.

^d^ “Enhanced” refers to an enhancement in health behaviors, including transitions from poor to moderate, poor to good, or moderate to good.

^e^ “Stable (good)” refers to individuals whose health behaviors remained moderate or good from pre‐ to post‐assessment (i.e., moderate–moderate or good‐good).

^f^ Participants categorized based on BMI (kg/m^2^) and waist circumference (cm)—poor: BMI < 19.0 or BMI ≥ 23.0 with waist circumference ≥ 90 (men) or ≥ 80 (women); moderate: BMI < 19.0 or BMI ≥ 23.0 with waist circumference < 90 (men) or < 80 (women); good: BMI between 19.0 and 23.0 with waist circumference < 90 (men) or < 80 (women).

^g^ Participants categorized based on the number of days of moderate to vigorous physical activity per week—poor: 0 days; moderate: 1–4 days; good group: ≥ 5 days.

^h^ Participants categorized based on the total alcohol consumption—poor: heavy drinkers (men: ≥ 2 drink/day, women: ≥ 1 drink/day); moderate: light drinkers (men: < 2 drink/day, women: < 1 drink/day); good: nondrinkers.

^i^ Participants categorized based on the smoking status—poor: current smokers; moderate: former smokers; good: nonsmokers.

### 3.5 Paradoxical Associations Between BMI Changes and All‐Cause Death

**FIGURE 2:**
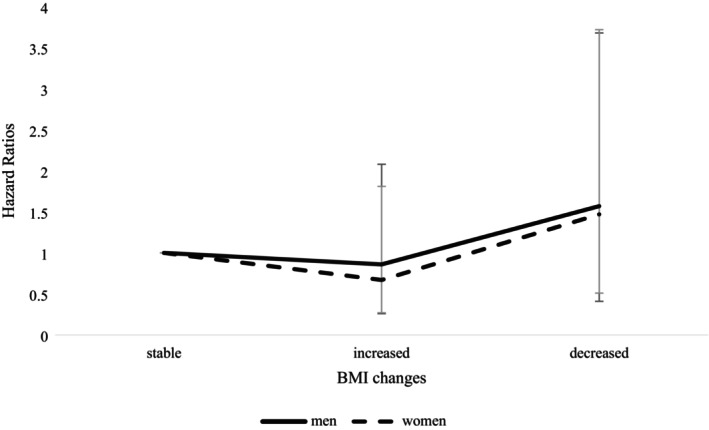
Hazard ratios for the risk of death from BMI changes among colorectal cancer survivor. BMI, body mass index. BMI (kg/m^2^) changes were calculated by subtracting pre‐diagnosis BMI from postdiagnosis BMI, where BMI change = 0 referred to stable, > 0 referred to increased BMI, and BMI change < 0 referred to decreased BMI. Lines are for 95% confidence intervals.

However, being or maintaining a healthy BMI range between 19.0 and 23.0 with waist circumference less than 90 cm (men) or less than 80 cm (women) was related to a greater risk, especially among those who were diagnosed with stage III cancer (enhanced: aHR = 1.75, 95% CI = 1.03–2.99; stable good: aHR = 1.55, 95% CI = 0.98–2.48), as shown in Table 4. Figure 2 further depicts the HRs for all‐cause death from BMI changes among CRC survivors, clearly showing the impact of BMI independently rather than in combination with waist circumference. BMI changes were defined as the difference between postdiagnosis and prediagnosis BMI, with positive values indicating an increase and negative values indicating a decrease in BMI. We therefore observed that an increase in BMI was associated with cancer prognosis, while a decrease in BMI was associated with increased risk of death. Specifically, using BMI < 21 as the reference group, the decreased risk of death was observed with increasing BMI among men. Meanwhile, the lowest risk was observed in the BMI range of 23–25, with the subsequent increase in risk beyond this range in women (Figure S1).

## 4 Discussion

The current retrospective cohort study provides notable findings about the effects of postdiagnosis health behaviors or their changing status on prognosis, underscoring the importance of physical activity and smoking cessation after CRC diagnosis for longer survival. Engaging in physical activity at least once per week after cancer diagnosis or maintaining such activity consistently throughout life (both before and after diagnosis) was associated with reduced mortality, particularly among those who were diagnosed with stage I or III CRC. In addition, the associations between postdiagnosis smoking status and mortality were not statistically significant; an overall trend suggested a lower risk of mortality compared to the reference group. Furthermore, extending the findings from the postdiagnosis status to behavioral changes, the study shows that individuals who consistently refrained from smoking before and after diagnosis had lower aHRs not only compared to the reference group but also to those in the enhanced group who reduced smoking after diagnosis. This highlights smoking as an important prognostic factor in CRC outcomes, especially not to smoke throughout the whole lifetime. Paradoxically, lowering BMI or waist circumference to meet the recommendations was related to increased all‐cause death, and the association was more prominent among those diagnosed with stage III CRC.

A previous meta‐analysis provided evidence that engaging in physical activity, both prior to and following a diagnosis of CRC, is related to lower risks of overall and cancer‐specific deaths [23]. The CUP Global study further recommended recreational physical activity for reducing all‐cause death and recurrence [24]. Another study reported that a low level of physical activity led to an extended postoperative hospital stay among CRC patients [25], and regular physical activity—regardless of when it was performed—reduced the risk of ischemic stroke among CRC patients [26]. This result expanded to Asian regions, as a recent study in Korea also reported that postdiagnosis physical activity was also associated with a reduced risk of death among cancer patients [12, 27]. However, whether the postdiagnosis physical activity impacts CRC independently of patients' prediagnosis activity levels remains uncertain [23]. In this context, the current study aimed to distinguish the effects of physical activity levels before and after cancer diagnosis by treating behavioral changes as an exposure variable, and revealed a decreased risk of death among individuals who engaged in physical activity either after their cancer diagnosis or consistently throughout their lifetime.

With regard to the smoking status, the results showing the clear negative prognostic effects of pre‐ or postdiagnosis smoking were mostly specific to lung cancer patients [28]. Although few studies have reported CRC‐specific outcomes, the results were usually not statistically powerful enough. For example, the Cancer Care Outcomes Research and Surveillance Consortium (CanCORS) study revealed that individuals who smoked at the time of their cancer diagnosis had lower survival rates than nonsmokers, but relatively small numbers of ever‐smokers in the CRC cohort were reported as the limitation [29]. Another Korean study suggested that the risk of smoking increased the risk of death among CRC patients, noting that the risk was even greater for those who smoked prior to their cancer diagnosis than for those who smoked postdiagnosis [30]. The current study also revealed that CRC patients who either quit or started smoking after cancer diagnosis had better survival rates than an association between increased mortality and smoking, not limited to postdiagnosis smoking but also among those who changed their behavior to quit smoking or start smoking, compared with individuals who smoked consistently both before and after the diagnosis, but statistical significance was observed only in the group diagnosed with CRC at stage II–III.

BMI or waist circumference and its effects on prognosis are highly related to the clinical status of CRC patients, and the results are highly inconsistent in the systematic review as follows [12, 31]; BMI and waist circumference decrease during surgery and increase again when patients are treated with chemotherapy, which makes it difficult to understand their effects on prognosis in CRC patients [32]. A previous systematic review revealed that being underweight (BMI < 18.5 kg/m^2^) or obese (BMI > 35 kg/m^2^) during surgery had disadvantages, whereas being overweight (BMI 25–30 kg/m^2^) improved survival outcomes [31]. However, other studies have indicated that CRC patients with a healthy weight presented a greater risk of death than those who were overweight after diagnosis [33, 34]. The current study investigated how close or distant the weight is from the recommended range and did not distinguish between weight gain and weight loss, potentially failing to clearly represent the relationship between body weight and prognosis. The relationships between BMI, categorized as increasing, maintaining, or decreasing, and all‐cause death are depicted in Figure 2. Furthermore, considering visceral fat measurements for future studies could provide more evidence, as suggested by the TOSCA trial, which revealed a trend toward better survival among stage II/III CRC survivors treated with adjuvant chemotherapy, suggesting the importance of considering visceral fat [35].

Accumulating evidence from previous studies, study results consistently demonstrated that adherence to WCRF/AICR recommendations is associated with improved survival among CRC patients [36, 37, 38]. Notably, physical activity emerges as the most robust factor, repeatedly related to enhanced survival across different populations and age groups, while body weight remains complex [38]. A previous study in Melbourne also suggested that prediagnosis lifestyle factors, including physical activity, waist circumference, and smoking, were significantly associated with survival outcomes in CRC patients, where the outcomes were not differed by microsatellite instability status [39]. In addition, CRC survivors made behavioral changes after their cancer diagnosis in an attempt to improve their lifestyle, as also demonstrated in this study [7, 40]. This suggests a need for appropriate guidelines for cancer patients. This underscores the need for clear, evidence‐based guidelines that patients can realistically follow. In this context, the American Cancer Society's nutrition and physical activity guideline for cancer survivors provides practical recommendations to support long‐term health and improve prognosis [41].

As a large‐scale retrospective cohort study, the current study included participants from a nationally representative database, making the data highly reliable and accurate. The population of the current study included the registered cancer patients, which is likely to address the limitations inherent in identifying cancer patients based solely on existing claims data [13]. Previous studies had the possibility of miscoding, since disease diagnoses might not accurately reflect patients' medical conditions as the researchers had to establish their own operational definition [42]. In addition, unlike prior studies conducted in Korea, which could not consider cancer stage [43], the present study incorporates stratification based on AJCC‐7 staging [19]. By integrating this clinical staging, the study offers a more refined understanding of postdiagnosis behaviors and survival outcomes [12]. This approach helps minimize the potential confounding effects of cancer stage on all‐cause death among CRC survivors [21].

Despite its strengths, this study has several limitations. First, the relatively short follow‐up time might not have been adequate to fully capture the potential impact of postdiagnosis behaviors on survival. Second, some details were not captured fully from the database. For example, the dates of health check‐up examinations were only available by year, while those of cancer diagnosis and death were provided by month. To address this, we assumed January as the month of health check‐up for all participants in the main analysis. Additionally, BMI and waist circumference were provided in coarse units of 1 kg/m^2^ and 10 cm only, limiting categorical classification. Ethanol intake by alcoholic beverage type became measurable from 2018, whereas earlier estimates relied on average consumption responses. Another limitation of the study is the absence of treatment‐related information. Although treatment was not directly considered in the analysis, subgroup analysis by cancer stage may reflect treatment patterns as treatment guidelines are generally determined by cancer stage [44, 45]. In addition, the study exclusively included individuals who underwent pre and postdiagnosis health check‐up examinations. When compared to the non‐screened population, the study population demonstrated distinct baseline characteristics—specifically, they tended to be younger, more likely male, and present with milder cases. This selection may limit the generalizability of the findings to broader colorectal cancer populations. When comparing the pre and postdiagnosis health behaviors, this study only examined the time points closest to the diagnosis—one before and after—which may not fully capture the time‐varying effects. Moreover, behavioral data were obtained through self‐reported questionnaires, which may introduce recall bias, potentially affecting the accuracy of exposure assessment. Finally, the study could not find any robust association between health behaviors and prognosis among early‐onset CRC patients due to the limited sample size.

## 5 Conclusions

This large‐scale retrospective cohort study highlights the significant benefits of postdiagnosis behavioral patterns in CRC survivors. Regular physical activity was associated with reduced all‐cause mortality, particularly among individuals diagnosed with CRC at stage I or III. In addition, a consistent trend indicated lower risk of death among those who did not smoke, especially when abstinence was maintained both before and after diagnosis. These findings underscore the importance of lifelong smoking avoidance as a key factor in improving CRC outcomes. Paradoxically, efforts to adjust BMI or waist circumference toward recommended levels were associated with increased mortality, with their potential relationship with other clinical status such as comorbidities, disease progression, and postsurgical outcomes. These results underscore that behavioral factors, especially physical activity and smoking, play a critical role in CRC prognosis and should be considered in survivorship care strategies.

## Author Contributions

**Donghyun Won:** conceptualization (supporting), data curation (lead), formal analysis (lead), investigation (equal), project administration (lead), software (lead), visualization (lead), writing – original draft (lead). **Ji Yoon Baek:** methodology (equal), validation (equal). **Ji Won Park:** validation (equal). **Jeeyoo Lee:** methodology (equal), validation (equal). **Sooyoung Cho:** writing – review and editing (lead). **Aesun Shin:** conceptualization (lead), funding acquisition (lead), supervision (lead), writing – review and editing (lead).

## Funding

This work was supported by a National Research Foundation of Korea (NRF) grant funded by the Korean government (MSIT) (No. 2022R1A2C1004608).

## Ethics Statement

The study was approved by the Institutional Review Boards of Seoul National University Hospital (IRB no. E‐2312‐132‐1496), and the procedures followed were in accordance with the ethical standards of the Helsinki Declaration.

## Consent

Informed consent was waived by the Institutional Review Board due to the retrospective nature of the study and the use of anonymized administrative data. All data were accessed in accordance with relevant data protection and privacy regulations.

## Conflicts of Interest

The authors declare no conflicts of interest.

## Supporting information


**Data S1:** Supplementary Information.

## Data Availability

The author (A.S.) has full access to all the data in the study and takes responsibility for the integrity of the data and accuracy of the data analysis. The data can be accessed by application to the Korea National Cancer Center (https://www.cancerdata.re.kr/).
