# Supplementary material for: Impact of Postdiagnosis Health Behaviors and Behavior Changes on Prognosis in Colorectal Cancer Patients: Evidence From Real‐World Data
Source: Cancer Med. 2025 Dec 21;14(24):e71461. doi: 10.1002/cam4.71461 (PMC12718552; doi:10.1002/cam4.71461)
Supplement: Supplementary file 1 — Data S1: Supplementary Information. [file CAM4-14-e71461-s001.docx]

## *Supplement Table 1. Cox proportional hazard models for the associations between postdiagnosis health behaviors and the risk of death by cancer stage and age group*

|  | **Stage I (N=2,685)** | | | **Stage II (N=2,087)** | | | **Stage III (N=2,250)** | | |
| --- | --- | --- | --- | --- | --- | --- | --- | --- | --- |
| **Group** | **Poor** | **Moderate** | **Good** | **Poor** | **Moderate** | **Good** | **Poor** | **Moderate** | **Good** |
| **Age <50 (N=750)** | | | | | | | | | |
| **Be a healthy weight^2^** | |  |  |  |  |  |  |  |  |
| N | 56 | 93 | 93 | 65 | 81 | 74 | 83 | 95 | 110 |
| Person-years | 31.7 | 56.9 | 61.9 | 39.8 | 49.2 | 48.7 | 45.8 | 56.1 | 63.7 |
| No. of deaths | 1 | 0 | 2 | 1 | 1 | 3 | 4 | 4 | 7 |
| HR 95% CI) | Ref. | NE | 0.89 (0.01-10.09) | Ref. | 0.90 (0.01-14.40) | 2.71 (0.28-26.16) | Ref. | 0.80 (0.20-3.19) | 1.24 (0.36-4.24) |
| aHR (95% CI) | Ref. | NE | NE | Ref. | 0.65 (0.03-12.24) | 1.42 (0.09-21.72) | Ref. | 1.29 (0.29-5.69) | 0.90 (0.24-3.42) |
| **Be physically active^3^** | |  |  |  |  |  |  |  |  |
| N | 74 | 148 | 20 | 71 | 137 | 12 | 83 | 184 | 21 |
| Person-years | 44.8 | 95.4 | 10.3 | 40.9 | 87.1 | 9.6 | 52.5 | 100.3 | 12.8 |
| No. of deaths | 1 | 2 | 0 | 2 | 3 | 0 | 8 | 6 | 1 |
| HR 95% CI) | Ref. | 0.98 (0.09-10.83) | NE | Ref. | 0.73 (0.12-4.38) | NE | Ref. | 0.39 (0.14-1.13) | 0.49 (0.06-3.91) |
| aHR (95% CI) | Ref. | NE | NE | Ref. | 1.54 (0.17-13.95) | NE | Ref. | 0.84 (0.26-2.71) | 0.65 (0.07-6.17) |
| **Limit alcohol consumption^4^** | |  |  |  |  |  |  |  |  |
| N | 46 | 75 | 121 | 22 | 71 | 127 | 28 | 62 | 198 |
| Person-years | 31.0 | 38.6 | 80.9 | 10.8 | 42.2 | 84.6 | 14.3 | 31.5 | 119.8 |
| No. of deaths | 0 | 0 | 3 | 0 | 1 | 4 | 0 | 0 | 15 |
| HR (95% CI) | Ref. | NE | NE | Ref. | NE | NE | Ref. | NE | NE |
| aHR (95% CI) | Ref. | NE | NE | Ref. | NE | NE | Ref. | NE | NE |
| **Quit smoking^5^** | |  |  |  |  |  |  |  |  |
| N | 30 | 91 | 121 | 18 | 74 | 128 | 17 | 97 | 174 |
| Person-years | 26.4 | 52.9 | 71.2 | 15.4 | 46.3 | 75.8 | 14.8 | 52.6 | 98.3 |
| No. of deaths | 0 | 0 | 3 | 0 | 2 | 3 | 0 | 3 | 12 |
| HR (95% CI) | Ref. | NE | NE | Ref. | NE | NE | Ref. | NE | NE |
| aHR (95% CI) | Ref. | NE | NE | Ref. | NE | NE | Ref. | NE | NE |
| **Age ≥50 (N=6,272)** | | | | | | | | | |
| **Be a healthy weight^2^** | |  |  |  |  |  |  |  |  |
| N | 979 | 778 | 686 | 720 | 567 | 580 | 759 | 586 | 617 |
| Person-years | 606.6 | 360.7 | 431.4 | 445.4 | 360.7 | 372.5 | 486.8 | 348.9 | 361.6 |
| No. of deaths | 42 | 37 | 45 | 54 | 38 | 77 | 76 | 69 | 105 |
| HR 95% CI) | Ref. | 1.05 (0.67-1.63) | 1.48 (0.97-2.26) | Ref. | 0.87 (0.57-1.31) | 1.72 (1.21-2.43) | Ref. | 1.27 (0.92-1.76) | 1.86 (1.39-2.50) |
| aHR (95% CI) | Ref. | 0.91 (0.58-1.44) | 1.36 (0.88-2.10) | Ref. | 0.82 (0.54-1.26) | 1.55 (1.09-2.21) | Ref. | 1.29 (0.92-1.80) | 1.78 (1.31-2.42) |
| **Be physically active^3^** | |  |  |  |  |  |  |  |  |
| N | 1,043 | 1,256 | 144 | 851 | 891 | 125 | 835 | 989 | 138 |
| Person-years | 742.8 | 699.3 | 101.9 | 591.5 | 501.3 | 85.8 | 548.9 | 552.4 | 96.0 |
| No. of deaths | 81 | 35 | 8 | 104 | 52 | 13 | 153 | 77 | 20 |
| HR 95% CI) | Ref. | **0.46 (0.31-0.68)** | 0.72 (0.35-1.49) | Ref. | **0.57 (0.41-0.80)** | 0.84 (0.47-1.49) | Ref. | **0.50 (0.38-0.66)** | 0.76 (0.47-1.21) |
| aHR (95% CI) | Ref. | **0.45 (0.30-0.67)** | 0.62 (0.30-1.28) | Ref. | **0.61 (0.43-0.85)** | 0.89 (0.50-1.59) | Ref. | **0.53 (0.40-0.70)** | 0.81 (0.51-1.31) |
| **Limit alcohol consumption^4^** | |  |  |  |  |  |  |  |  |
| N | 265 | 549 | 1,629 | 148 | 295 | 1,424 | 112 | 282 | 1,568 |
| Person-years | 157.2 | 292.2 | 1,094.8 | 83.7 | 150.5 | 944.4 | 47.0 | 141.8 | 1,008.5 |
| No. of deaths | 11 | 16 | 97 | 12 | 15 | 142 | 11 | 16 | 223 |
| HR 95% CI) | Ref. | 0.80 (0.37-1.72) | 1.27 (0.68-2.37) | Ref. | 0.68 (0.32-1.45) | 1.03 (0.57-1.85) | Ref. | 0.48 (0.22-1.03) | 0.94 (0.52-1.73) |
| aHR (95% CI) | Ref. | 0.98 (0.45-2.12) | 1.71 (0.91-3.24) | Ref. | 0.73 (0.34-1.56) | 1.21 (0.66-2.22) | Ref. | 0.52 (0.24-1.12) | 0.85 (0.46-1.58) |
| **Quit smoking^5^** |  |  |  |  |  |  |  |  |  |
| N | 163 | 818 | 1,462 | 100 | 605 | 1,162 | 99 | 655 | 1,208 |
| Person-years | 150.1 | 484.3 | 909.8 | 90.6 | 350.1 | 737.9 | 79.1 | 386.6 | 731.7 |
| No. of deaths | 19 | 34 | 71 | 15 | 58 | 96 | 22 | 71 | 157 |
| HR (95% CI) | Ref. | 0.57 (0.33-1.00) | 0.63 (0.38-1.05) | Ref. | 1.04 (0.59-1.85) | 0.82 (0.48-1.42) | Ref. | 0.66 (0.41-1.06) | 0.77 (0.49-1.21) |
| aHR (95% CI) | Ref. | 0.62 (0.35-1.10) | 0.96 (0.55-1.66) | Ref. | 1.00 (0.56-1.78) | 0.92 (0.51-1.66) | Ref. | 0.66 (0.41-1.07) | 0.84 (0.52-1.35) |
| Abbreviation: N/No., the number of corresponding cases; Ref., Reference; aHR, adjusted Hazard Ratios; CI, Confidence Interval; NE, not estimated; BMI, Body Mass Index.  1 The models were adjusted for sex (men or women), year post-diagnosis, insurance type (local insured, employee insured or medical-aid beneficiary), income level (decile to tertile; tertile 1 (0,1,2,3,4), tertile 2 (5,6,7), tertile 3 (8,9,10) or missing), and residence (Seoul, metropolitan cities or others).  2 Participants categorized based on BMI (kg/m²) and waist circumference (cm) - Poor: BMI <19.0 or BMI ≥23.0 with waist circumference ≥90 (men) or ≥80 (women); Moderate: BMI <19.0 or BMI ≥23.0 with waist circumference <90 (men) or <80 (women); Good: BMI between 19.0 and 23.0 with waist circumference <90 (men) or <80 (women).  3 Participants categorized based on the number of days of moderate to vigorous physical activity per week - Poor: 0 days; Moderate: 1-4 days; Good group: ≥5 days.  4 Participants categorized based on the total alcohol consumption - Poor: heavy drinkers (men: ≥2 drink/day, women: ≥1 drink/day); Moderate: light drinkers (men: <2 drink/day, women: <1 drink/day); Good: non-drinkers.  5 Participants categorized based on by the smoking status - Poor: current smokers; Moderate: former smokers; Good: non-smokers. | | | | | | | | | |

## *Supplement Table 2. Cox proportional hazard models for the associations between postdiagnosis health behaviors and the risk of death by cancer stage and cancer sites*

|  | **Stage I (N=2,684)** | | | **Stage II (N=2,085)** | | | **Stage III (N=2,246)** | | |
| --- | --- | --- | --- | --- | --- | --- | --- | --- | --- |
| **Group** | **Poor** | **Moderate** | **Good** | **Poor** | **Moderate** | **Good** | **Poor** | **Moderate** | **Good** |
| **Colon (N=4,251)^2^** | | | | | | | | | |
| **Be a healthy weight^3^** | |  |  |  |  |  |  |  |  |
| N | 646 | 527 | 414 | 549 | 450 | 422 | 510 | 373 | 360 |
| Person-years | 410.3 | 326.1 | 260.3 | 339.7 | 294.4 | 275.9 | 313.4 | 217.2 | 213.4 |
| No. of deaths | 22 | 23 | 14 | 31 | 30 | 51 | 47 | 37 | 57 |
| HR 95% CI) | Ref. | 1.31 (0.73-2.35) | 0.97 (0.50-1.90) | Ref. | 1.11 (0.67-1.84) | **2.03 (1.30-3.17)** | Ref. | 1.14 (0.74-1.75) | **1.78 (1.21-2.62)** |
| aHR (95% CI) | Ref. | 1.35 (0.73-2.49) | 1.04 (0.52-2.07) | Ref. | 1.03 (0.61-1.72) | **1.74 (1.09-2.78)** | Ref. | 1.36 (0.88-2.12) | **1.76 (1.19-2.62)** |
| **Be physically active^4^** | |  |  |  |  |  |  |  |  |
| N | 654 | 844 | 89 | 634 | 696 | 91 | 515 | 645 | 83 |
| Person-years | 458.3 | 481.8 | 56.6 | 440.1 | 409.9 | 60.0 | 337.5 | 351.3 | 55.2 |
| No. of deaths | 42 | 14 | 3 | 65 | 40 | 7 | 85 | 46 | 10 |
| HR 95% CI) | Ref. | **0.31 (0.17-0.57)** | 0.59 (0.18-1.92) | Ref. | **0.64 (0.43-0.94)** | 0.80 (0.37-1.75) | Ref. | **0.52 (0.36-0.74)** | 0.73 (0.38-1.41) |
| aHR (95% CI) | Ref. | **0.38 (0.20-0.71)** | 0.53 (0.16-1.73) | Ref. | 0.90 (0.60-1.35) | 1.21 (0.55-2.68) | Ref. | **0.66 (0.45-0.96)** | 1.07 (0.54-2.12) |
| **Limit alcohol consumption^5^** | |  |  |  |  |  |  |  |  |
| N | 190 | 380 | 1,017 | 121 | 239 | 1,061 | 69 | 195 | 979 |
| Person-years | 115.7 | 201.3 | 679.8 | 66.0 | 137.0 | 707.0 | 31.5 | 94.8 | 617.7 |
| No. of deaths | 6 | 7 | 46 | 7 | 9 | 96 | 8 | 6 | 127 |
| HR (95% CI) | Ref. | 0.70 (0.23-2.08) | 1.33 (0.57-3.12) | Ref. | 0.63 (0.24-1.70) | 1.31 (0.61-2.82) | Ref. | **0.25 (0.09-0.72)** | 0.82 (0.40-1.68) |
| aHR (95% CI) | Ref. | 0.52 (0.17-1.59) | 0.95 (0.38-2.33) | Ref. | 0.70 (0.26-1.89) | 1.18 (0.53-2.62) | Ref. | **0.21 (0.07-0.62)** | 0.55 (0.27-1.16) |
| **Quit smoking^6^** | |  |  |  |  |  |  |  |  |
| N | 113 | 522 | 952 | 70 | 434 | 917 | 53 | 395 | 795 |
| Person-years | 104.4 | 312.9 | 579.3 | 63.6 | 257.3 | 589.1 | 45.8 | 221.4 | 476.8 |
| No. of deaths | 8 | 16 | 35 | 8 | 37 | 67 | 10 | 36 | 95 |
| HR (95% CI) | Ref. | 0.70 (0.30-1.64) | 0.83 (0.38-1.79) | Ref. | 1.18 (0.55-2.54) | 0.94 (0.45-1.95) | Ref. | 0.74 (0.37-1.50) | 0.92 (0.48-1.76) |
| aHR (95% CI) | Ref. | 0.59 (0.25-1.42) | 0.55 (0.23-1.31) | Ref. | 1.01 (0.47-2.18) | 0.77 (0.35-1.69) | Ref. | 0.56 (0.28-1.15) | 0.69 (0.34-1.41) |
| **Rectum (N=2,764)^2^** | | | | | | | | | |
| **Be a healthy weight^3^** | |  |  |  |  |  |  |  |  |
| N | 389 | 344 | 364 | 235 | 198 | 231 | 329 | 307 | 367 |
| Person-years | 227.9 | 236.9 | 231.7 | 145.4 | 115.4 | 145.2 | 218.8 | 186.9 | 211.8 |
| No. of deaths | 21 | 14 | 33 | 24 | 9 | 29 | 33 | 36 | 55 |
| HR 95% CI) | Ref. | 0.64 (0.32-1.25) | 1.53 (0.88-2.65) | Ref. | 0.47 (0.22-1.02) | 1.20 (0.70-2.06) | Ref. | 1.28 (0.80-2.06) | **1.73 (1.12-2.67)** |
| aHR (95% CI) | Ref. | 0.65 (0.32-1.32) | 1.12 (0.80-2.53) | Ref. | 0.52 (0.24-1.14) | 1.18 (0.68-2.04) | Ref. | 1.50 (0.91-2.46) | **1.72 (1.09-2.71)** |
| **Be physically active^4^** | |  |  |  |  |  |  |  |  |
| N | 463 | 559 | 75 | 287 | 331 | 46 | 401 | 526 | 76 |
| Person-years | 329.4 | 311.5 | 55.6 | 192.3 | 178.4 | 35.3 | 263.5 | 300.3 | 53.7 |
| No. of deaths | 40 | 23 | 5 | 41 | 15 | 6 | 76 | 37 | 11 |
| HR 95% CI) | Ref. | 0.62 (0.37-1.04) | 0.74 (0.29-1.88) | Ref. | **0.39 (0.21-0.70)** | 0.77 (0.33-1.84) | Ref. | **0.43 (0.29-0.64)** | 0.72 (0.38-1.35) |
| aHR (95% CI) | Ref. | 0.73 (0.43-1.25) | 0.61 (0.24-1.57) | Ref. | **0.50 (0.27-0.93)** | 0.90 (0.36-2.22) | Ref. | **0.62 (0.41-0.93)** | 0.96 (0.50-1.83) |
| **Limit alcohol consumption^5^** | |  |  |  |  |  |  |  |  |
| N | 121 | 243 | 733 | 49 | 127 | 488 | 71 | 148 | 784 |
| Person-years | 72.5 | 128.1 | 495.9 | 28.5 | 55.7 | 321.8 | 29.8 | 78.4 | 509.3 |
| No. of deaths | 5 | 9 | 54 | 5 | 7 | 50 | 3 | 10 | 111 |
| HR 95% CI) | Ref. | 1.02 (0.34-3.03) | 1.56 (0.63-3.91) | Ref. | 0.69 (0.22-2.16) | 0.86 (0.34-2.15) | Ref. | 1.24 (0.34-4.51) | 2.11 (0.67-6.64) |
| aHR (95% CI) | Ref. | 1.03 (0.34-3.15) | 1.45 (0.55-3.80) | Ref. | 0.58 (0.18-1.92) | 0.63 (0.24-1.69) | Ref. | 1.34 (0.37-4.91) | 1.76 (0.55-5.63) |
| **Quit smoking^6^** |  |  |  |  |  |  |  |  |  |
| N | 79 | 387 | 631 | 48 | 245 | 371 | 63 | 357 | 583 |
| Person-years | 70.7 | 224.3 | 401.6 | 42.4 | 139.1 | 224.5 | 48.0 | 217.8 | 351.8 |
| No. of deaths | 11 | 18 | 39 | 7 | 23 | 32 | 12 | 38 | 74 |
| HR (95% CI) | Ref. | 0.52 (0.25-1.11) | 0.63 (0.32-1.23) | Ref. | 1.01 (0.43-2.37) | 0.88 (0.39-1.99) | Ref. | 0.70 (0.37-1.35) | 0.85 (0.46-1.57) |
| aHR (95% CI) | Ref. | 0.51 (0.24-1.11) | 0.76 (0.36-1.59) | Ref. | 0.81 (0.34-1.95) | 0.79 (0.32-1.95) | Ref. | 0.63 (0.33-1.23) | 0.70 (0.36-1.35) |
| Abbreviation: N/No., the number of corresponding cases; Ref., Reference; aHR, adjusted Hazard Ratios; CI, Confidence Interval; NE, not estimated; BMI, Body Mass Index.  1 The models were adjusted for age, sex (men or women), year post-diagnosis, insurance type (local insured, employee insured or medical-aid beneficiary), income level (decile to tertile; tertile 1 (0,1,2,3,4), tertile 2 (5,6,7), tertile 3 (8,9,10) or missing), and residence (Seoul, metropolitan cities or others).  2 Colon cancer was defined as ICD-O-3 codes C18.0 and C18.2-C18.9. Rectum cancer was defined as ICD-O-3 codes C19.9 and C20.9. Cases of multiple primary cancers (n=7) were excluded from the analysis.  3 Participants categorized based on BMI (kg/m²) and waist circumference (cm) - Poor: BMI <19.0 or BMI ≥23.0 with waist circumference ≥90 (men) or ≥80 (women); Moderate: BMI <19.0 or BMI ≥23.0 with waist circumference <90 (men) or <80 (women); Good: BMI between 19.0 and 23.0 with waist circumference <90 (men) or <80 (women).  4 Participants categorized based on the number of days of moderate to vigorous physical activity per week - Poor: 0 days; Moderate: 1-4 days; Good group: ≥5 days.  5 Participants categorized based on the total alcohol consumption - Poor: heavy drinkers (men: ≥2 drink/day, women: ≥1 drink/day); Moderate: light drinkers (men: <2 drink/day, women: <1 drink/day); Good: non-drinkers.  6 Participants categorized based on by the smoking status - Poor: current smokers; Moderate: former smokers; Good: non-smokers. | | | | | | | | | |

## *Supplement Table 3. Cox proportional hazard models for the association between behavior changes and the risk of death by cancer stage and age group*

|  | **Stage I (n=1,716)** | | | | | | | **Stage II (n=1,246)** | | | | | | | | **Stage III (n=1,333)** | | | | | | |
| --- | --- | --- | --- | --- | --- | --- | --- | --- | --- | --- | --- | --- | --- | --- | --- | --- | --- | --- | --- | --- | --- | --- |
| **Behavior Changes** | **Unchanged (poor)^2^** | **Worsen^3^** | | **Enhanced^4^** | | **Stable (good)^5^** | | **Unchanged (poor)^2^** | | **Worsen^3^** | | **Enhanced^4^** | | **Stable (good)^5^** | | **Unchanged (poor)^2^** | | **Worsen^3^** | | **Enhanced^4^** | | **Stable (good)^5^** |
| **Age <50 (N=375)** | | | | | | | | | | | | | | | | | | | | | | |
| **Be a healthy weight^6^** | |  | |  | |  | |  | |  | |  | |  | |  | |  | |  | |  |
| N | 11 | 22 | | 20 | | 71 | | 22 | | 21 | | 16 | | 51 | | 20 | | 27 | | 22 | | 72 |
| Person-years | 6.4 | 9.8 | | 10.8 | | 32.8 | | 11.1 | | 10.3 | | 11.0 | | 28.3 | | 8.9 | | 10.6 | | 8.7 | | 35.0 |
| No. of deaths | 0 | 0 | | 0 | | 0 | | 0 | | 1 | | 2 | | 2 | | 0 | | 1 | | 2 | | 1 |
| HR (95% CI) | Ref. | NE | | NE | | NE | | Ref. | | NE | | NE | | NE | | Ref. | | NE | | NE | | NE |
| aHR (95% CI) | Ref. | NE | | NE | | NE | | Ref. | | NE | | NE | | NE | | Ref. | | NE | | NE | | NE |
| **Be physically active^7^** | |  | |  | |  | |  | |  | |  | |  | |  | |  | |  | |  |
| N | 25 | 13 | | 34 | | 52 | | 22 | | 19 | | 30 | | 39 | | 19 | | 19 | | 49 | | 54 |
| Person-years | 10.3 | 6.8 | | 19.3 | | 23.6 | | 10.4 | | 6.8 | | 19.3 | | 24.3 | | 8.0 | | 12.3 | | 16.6 | | 26.3 |
| No. of deaths | 0 | 0 | | 0 | | 0 | | 0 | | 3 | | 0 | | 2 | | 1 | | 0 | | 1 | | 2 |
| HR  (95% CI) | Ref. | NE | | NE | | NE | | Ref. | | NE | | NE | | NE | | Ref. | | NE | | 0.73  (0.04-12.82) | | 0.76  (0.07-8.55) |
| aHR  (95% CI) | Ref. | NE | | NE | | NE | | Ref. | | NE | | NE | | NE | | Ref. | | NE | | 0.36  (0.01-14.27) | | 4.73  (0.10-215.16) |
| **Limit alcohol consumption^8^** | | |  | |  | |  | |  | |  | |  | |  | |  | |  | |  | |
| N | 15 | 17 | | 40 | | 52 | | 7 | | 12 | | 37 | | 54 | | 11 | | 10 | | 50 | | 70 |
| Person-years | 6.9 | 7.4 | | 21.9 | | 23.6 | | 1.9 | | 6.8 | | 22.7 | | 29.4 | | 3.0 | | 3.8 | | 28.9 | | 27.5 |
| No. of deaths | 0 | 0 | | 0 | | 0 | | 0 | | 0 | | 2 | | 3 | | 0 | | 0 | | 4 | | 0 |
| HR (95% CI) | Ref. | NE | | NE | | NE | | Ref. | | NE | | NE | | NE | | Ref. | | NE | | NE | | NE |
| aHR (95% CI) | Ref. | NE | | NE | | NE | | Ref. | | NE | | NE | | NE | | Ref. | | NE | | NE | | NE |
| **Quit smoking^9^** | |  | |  | |  | |  | |  | |  | |  | |  | |  | |  | |  |
| N | 10 | 4 | | 45 | | 65 | | 7 | | 3 | | 29 | | 71 | | 4 | | 2 | | 44 | | 91 |
| Person-years | 6.9 | 1.8 | | 19.0 | | 32.1 | | 4.3 | | 1.3 | | 14.3 | | 40.8 | | 0.3 | | 0.7 | | 18.3 | | 43.9 |
| No. of deaths | 0 | 0 | | 0 | | 0 | | 0 | | 0 | | 1 | | 4 | | 0 | | 0 | | 1 | | 3 |
| HR (95% CI) | Ref. | NE | | NE | | NE | | Ref. | | NE | | NE | | NE | | Ref. | | NE | | NE | | NE |
| aHR (95% CI) | Ref. | NE | | NE | | NE | | Ref. | | NE | | NE | | NE | | Ref. | | NE | | NE | | NE |
| **Age** $\boldsymbol{\geq}$**50 (N=3,920)** | | | | | | | | | | | | | | | | | | | | | | |
| **Be a healthy weight^6^** | |  | |  | |  | |  | |  | |  | |  | |  | |  | |  | |  |
| N | 457 | 270 | | 244 | | 621 | | 328 | | 170 | | 184 | | 454 | | 314 | | 213 | | 214 | | 451 |
| Person-years | 252.5 | 127.3 | | 121.3 | | 336.1 | | 181.3 | | 83.9 | | 111.5 | | 239.8 | | 164.0 | | 107.0 | | 106.2 | | 230.3 |
| No. of deaths | 12 | 9 | | 8 | | 26 | | 25 | | 4 | | 15 | | 32 | | 27 | | 14 | | 26 | | 53 |
| HR  (95% CI) | Ref. | 1.53  (0.64-3.64) | | 1.38  (0.56-3.38) | | 1.62  (0.82-3.22) | | Ref. | | 0.35  (0.12-1.00) | | 0.99  (0.52-1.88) | | 0.97  (0.58-1.64) | | Ref. | | 0.80  (0.42-1.52) | | 1.48  (0.86-2.54) | | 1.41  (0.89-2.24) |
| aHR  (95% CI) | Ref. | 1.42  (0.59-3.42) | | 1.49  (0.59-3.71) | | 1.41  (0.69-2.91) | | Ref. | | 0.34  (0.12-0.99) | | 1.08  (0.56-2.05) | | 0.93  (0.54-1.59) | | Ref. | | 0.82  (0.43-1.58) | | 1.46  (0.84-2.53) | | 1.45  (0.90-2.33) |
| **Be physically active^7^** | |  | |  | |  | |  | |  | |  | |  | |  | |  | |  | |  |
| N | 432 | 275 | | 453 | | 432 | | 352 | | 186 | | 309 | | 289 | | 313 | | 203 | | 359 | | 317 |
| Person-years | 270.8 | 155.3 | | 205.8 | | 205.3 | | 214.7 | | 106.4 | | 155.7 | | 139.8 | | 174.6 | | 104.8 | | 176.3 | | 151.8 |
| No. of deaths | 26 | 13 | | 11 | | 5 | | 38 | | 11 | | 19 | | 8 | | 48 | | 32 | | **22** | | **18** |
| HR  (95% CI) | Ref. | 0.85  (0.44-1.66) | | 0.57  (0.28-1.15) | | **0.25**  **(0.10-0.66)** | | Ref. | | 0.58  (0.29-1.13) | | 0.67  (0.38-1.16) | | **0.32**  **(0.15-0.69)** | | Ref. | | 1.10  (0.70-1.72) | | **0.45**  **(0.27-0.75)** | | **0.43**  **(0.25-0.74)** |
| aHR  (95% CI) | Ref. | 0.84  (0.43-1.62) | | 0.52  (0.25-1.06) | | **0.25**  **(0.09-0.66)** | | Ref. | | 0.65  (0.33-1.29) | | 0.71  (0.40-1.24) | | **0.34**  **(0.16-0.74)** | | Ref. | | 1.11  (0.70-1.75) | | **0.49**  **(0.29-0.82)** | | **0.50**  **(0.29-0.88)** |
| **Limit alcohol consumption^8^** | | |  | |  | |  | |  | |  | |  | |  | |  | |  | |  | |
| N | 125 | 138 | | 381 | | 948 | | 68 | | 66 | | 308 | | 694 | | 45 | | 64 | | 390 | | 693 |
| Person-years | 53.6 | 55.4 | | 215.5 | | 512.7 | | 33.7 | | 29.4 | | 173.3 | | 380.1 | | 13.7 | | 22.5 | | 223.8 | | 347.6 |
| No. of deaths | 3 | 3 | | 13 | | 36 | | 6 | | 3 | | 21 | | 46 | | 1 | | 4 | | 36 | | 79 |
| HR  (95% CI) | Ref. | 0.99  (0.20-4.89) | | 1.07  (0.30-3.75) | | 1.23  (0.38-4.00) | | Ref. | | 0.56  (0.14-2.26) | | 0.68  (0.28-1.69) | | 0.69  (0.29-1.60) | | Ref. | | 2.36  (0.26-21.11) | | 2.21  (0.30-16.10) | | 3.11  (0.43-22.36) |
| aHR  (95% CI) | Ref. | 1.28  (0.25-6.42) | | 1.31  (0.37-4.67) | | 1.70  (0.51-5.64) | | Ref. | | 0.72  (0.18-2.92) | | 0.78  (0.31-1.97) | | 0.83  (0.33-2.04) | | Ref. | | 2.19  (0.24-19.75) | | 1.85  (0.25-13.62) | | 2.56  (0.35-18.76) |
| **Quit smoking^9^** | |  | |  | |  | |  | |  | |  | |  | |  | |  | |  | |  |
| N | 70 | 105 | | 349 | | 1,068 | | 40 | | 54 | | 253 | | 789 | | 53 | | 71 | | 261 | | 807 |
| Person-years | 54.8 | 53.5 | | 168.7 | | 560.2 | | 29.1 | | 27.5 | | 117.0 | | 442.9 | | 36.3 | | 43.2 | | 127.9 | | 400.1 |
| No. of deaths | 5 | 6 | | 12 | | 32 | | 10 | | 3 | | 14 | | 49 | | 9 | | 4 | | 31 | | 76 |
| HR  (95% CI) | Ref. | 1.29  (0.39-4.24) | | 0.83  (0.29-2.38) | | 0.65  (0.25-1.68) | | Ref. | | 0.31  (0.08-1.11) | | **0.34**  **(0.15-0.78)** | | **0.33**  **(0.17-0.64)** | | Ref. | | 0.37  (0.12-1.21) | | 0.97  (0.46-2.04) | | 0.76  (0.38-1.52) |
| aHR  (95% CI) | Ref. | 1.48  (0.45-4.89) | | 1.00  (0.34-2.90) | | 0.98  (0.36-2.67) | | Ref. | | **0.26**  **(0.07-0.95)** | | **0.30**  **(0.13-0.68)** | | **0.33**  **(0.16-0.69)** | | Ref. | | 0.35  (0.11-1.15) | | 1.02  (0.48-2.17) | | 0.67  (0.32-1.39) |
| Abbreviation: N/No., the number of corresponding cases; Ref., reference; aHR, adjusted Hazard Ratios; CI, Confidence Interval; NE, not estimated; BMI, Body Mass Index.  1 The models were adjusted for age, sex (men or women), year post-diagnosis, insurance type (local insured, employee insured or medical-aid beneficiary), income level (decile to tertile; tertile 1 (0,1,2,3,4), tertile 2 (5,6,7), tertile 3 (8,9,10) or missing), and residence (Seoul, metropolitan cities or others).  2 “Unchanged (poor)” refers to individuals whose health behaviors remained poor from pre- to post-assessment (i.e., poor–poor).  3 “Worsen” refers to a worsen in health behaviors, including transitions from moderate to poor, good to poor, or good to moderate.  4 “Enhanced” refers to an enhancement in health behaviors, including transitions from poor to moderate, poor to good, or moderate to good.  5 “Stable (good)” refers to individuals whose health behaviors remained moderate or good from pre- to post-assessment (i.e., moderate-moderate or good-good).  6 Participants categorized based on BMI (kg/m²) and waist circumference (cm) - Poor: BMI <19.0 or BMI ≥23.0 with waist circumference ≥90 (men) or ≥80 (women); Moderate: BMI <19.0 or BMI ≥23.0 with waist circumference <90 (men) or <80 (women); Good: BMI between 19.0 and 23.0 with waist circumference <90 (men) or <80 (women).  7 Participants categorized based on the number of days of moderate to vigorous physical activity per week - Poor: 0 days; Moderate: 1-4 days; Good group: ≥5 days.  8 Participants categorized based on the total alcohol consumption - Poor: heavy drinkers (men: ≥2 drink/day, women: ≥1 drink/day); Moderate: light drinkers (men: <2 drink/day, women: <1 drink/day); Good: non-drinkers.  9 Participants categorized based on the smoking status - Poor: current smokers; Moderate: former smokers; Good: non-smokers. | | | | | | | | | | | | | | | | | | | | | | |

## *Supplement Table 4. Cox proportional hazard models for the association between behavior changes and the risk of death by cancer stage and cancer sites*

|  | **Stage I (n=1,716)** | | | | | | | **Stage II (n=1,244)** | | | | | | | | **Stage III (n=1,329)** | | | | | | |
| --- | --- | --- | --- | --- | --- | --- | --- | --- | --- | --- | --- | --- | --- | --- | --- | --- | --- | --- | --- | --- | --- | --- |
| **Behavior Changes** | **Unchanged (poor)^2^** | **Worsen^3^** | | **Enhanced^4^** | | **Stable (good)^5^** | | **Unchanged (poor)^2^** | | **Worsen^3^** | | **Enhanced^4^** | | **Stable (good)^5^** | | **Unchanged (poor)^2^** | | **Worsen^3^** | | **Enhanced^4^** | | **Stable (good)^5^** |
| **Colon (N=2,648)^6^** | | | | | | | | | | | | | | | | | | | | | | |
| **Be a healthy weight^7^** | |  | |  | |  | |  | |  | |  | |  | |  | |  | |  | |  |
| N | 312 | 176 | | 169 | | 396 | | 246 | | 134 | | 133 | | 323 | | 213 | | 143 | | 128 | | 275 |
| Person-years | 180.6 | 83.9 | | 78.5 | | 208.6 | | 134.7 | | 66.4 | | 80.2 | | 184.0 | | 109.5 | | 71.3 | | 59.8 | | 141.8 |
| No. of deaths | 6 | 7 | | 4 | | 7 | | 12 | | 4 | | 12 | | 23 | | 19 | | 5 | | 17 | | 28 |
| HR  (95% CI) | Ref. | 2.60  (0.87-7.78) | | 1.57  (0.44-5.57) | | 1.00  (0.34-2.98) | | Ref. | | 0.69  (0.22-2.15) | | 1.71  (0.77-3.81) | | 1.41  (0.70-2.82) | | Ref. | | 0.41  (0.15-1.10) | | 1.64  (0.85-3.15) | | 1.15  (0.64-2.06) |
| aHR  (95% CI) | Ref. | 2.72  (0.90-8.23) | | 1.55  (0.42-5.72) | | 0.89  (0.28-2.81) | | Ref. | | 0.74  (0.24-2.33) | | 1.70  (0.75-3.89) | | 1.41  (0.68-2.91) | | Ref. | | 0.49  (0.18-1.34) | | 1.48  (0.75-2.89) | | 1.30  (0.71-2.37) |
| **Be physically active^8^** | |  | |  | |  | |  | |  | |  | |  | |  | |  | |  | |  |
| N | 280 | 179 | | 276 | | 318 | | 247 | | 146 | | 226 | | 217 | | 190 | | 133 | | 224 | | 212 |
| Person-years | 177.3 | 99.8 | | 133.5 | | 140.9 | | 152.5 | | 80.4 | | 120.8 | | 111.6 | | 110.6 | | 67.3 | | 101.6 | | 102.8 |
| No. of deaths | 13 | 6 | | 4 | | 1 | | 20 | | 9 | | 13 | | 9 | | 26 | | 21 | | 11 | | 11 |
| HR  (95% CI) | Ref. | 0.80  (0.30-2.11) | | 0.41  (0.13-1.25) | | **0.10**  **(0.01-0.74)** | | Ref. | | 0.85  (0.39-1.87) | | 0.83  (0.41-1.67) | | 0.62  (0.28-1.36) | | Ref. | | 1.33  (0.74-2.36) | | **0.46**  **(0.23-0.94)** | | **0.45**  **(0.22-0.92)** |
| aHR  (95% CI) | Ref. | 0.84  (0.31-2.29) | | 0.35  (0.11-1.14) | | **0.12**  **(0.02-0.95)** | | Ref. | | 0.95  (0.43-2.14) | | 1.15  (0.55-2.39) | | 0.85  (0.36-1.97) | | Ref. | | 1.53  (0.84-2.79) | | 0.60  (0.29-1.25) | | 0.69  (0.33-1.43) |
| **Limit alcohol consumption^9^** | | |  | |  | |  | |  | |  | |  | |  | |  | |  | |  | |
| N | 95 | 101 | | 244 | | 613 | | 50 | | 55 | | 213 | | 518 | | 26 | | 46 | | 232 | | 455 |
| Person-years | 43.0 | 43.9 | | 135.2 | | 329.5 | | 23.7 | | 27.3 | | 125.9 | | 288.4 | | 8.3 | | 18.3 | | 139.7 | | 216.1 |
| No. of deaths | 1 | 3 | | 6 | | 14 | | 2 | | 2 | | 15 | | 32 | | 1 | | 2 | | 23 | | 43 |
| HR  (95% CI) | Ref. | 3.02  (0.31-29.05) | | 1.91  (0.23-15.86) | | 1.83  (0.24-13.92) | | Ref. | | 0.90  (0.13-6.37) | | 1.43  (0.33-6.26) | | 1.33  (0.32-5.56) | | Ref. | | 0.85  (0.08-9.34) | | 1.25  (0.17-9.31) | | 1.55  (0.21-11.26) |
| aHR  (95% CI) | Ref. | 1.78  (0.18-17.61) | | 1.34  (0.16-11.39) | | 0.97  (0.12-7.91) | | Ref. | | 1.10  (0.15-8.18) | | 1.48  (0.33-6.60) | | 1.29  (0.29-5.77) | | Ref. | | 0.75  (0.07-8.74) | | 1.18  (0.15-9.12) | | 1.07  (0.14-8.38) |
| **Quit smoking^10^** | |  | |  | |  | |  | |  | |  | |  | |  | |  | |  | |  |
| N | 50 | 62 | | 236 | | 705 | | 29 | | 31 | | 175 | | 601 | | 27 | | 38 | | 164 | | 530 |
| Person-years | 41.7 | 31.5 | | 119.8 | | 358.7 | | 22.3 | | 15.5 | | 83.3 | | 344.1 | | 20.0 | | 25.7 | | 80.0 | | 256.7 |
| No. of deaths | 0 | 2 | | 6 | | 16 | | 6 | | 1 | | 9 | | 35 | | 4 | | 2 | | 17 | | 46 |
| HR  (95% CI) | Ref. | NE | | NE | | NE | | Ref. | | 0.24  (0.03-2.03) | | 0.42  (0.15-1.18) | | 0.40  (0.17-0.94) | | Ref. | | 0.38  (0.07-2.08) | | 1.06  (0.36-3.17) | | 0.92  (0.33-2.55) |
| aHR  (95% CI) | Ref. | NE | | NE | | NE | | Ref. | | 0.20  (0.02-1.73) | | 0.35  (0.12-1.03) | | **0.33**  **(0.12-0.86)** | | Ref. | | 0.23  (0.04-1.32) | | 1.05  (0.34-3.23) | | 0.71  (0.23-2.17) |
| **Rectum (N=1,641)^6^** | | | | | | | | | | | | | | | | | | | | | | |
| **Be a healthy weight^7^** | |  | |  | |  | |  | |  | |  | |  | |  | |  | |  | |  |
| N | 156 | 116 | | 95 | | 296 | | 103 | | 57 | | 67 | | 181 | | 120 | | 94 | | 108 | | 248 |
| Person-years | 78.3 | 53.2 | | 53.6 | | 160.3 | | 57.6 | | 27.8 | | 42.3 | | 84.1 | | 63.1 | | 45.3 | | 55.1 | | 123.5 |
| No. of deaths | 6 | 2 | | 4 | | 19 | | 13 | | 1 | | 5 | | 11 | | 8 | | 10 | | 11 | | 26 |
| HR  (95% CI) | Ref. | 0.51  (0.10-2.51) | | 0.91  (0.26-3.23) | | 1.53  (0.61-3.83) | | Ref. | | 0.15  (0.02-1.16) | | 0.53  (0.19-1.48) | | 0.57  (0.26-1.28) | | Ref. | | 1.71  (0.68-4.33) | | 1.55  (0.62-3.86) | | 1.65  (0.75-3.65) |
| aHR  (95% CI) | Ref. | 0.32  (0.06-1.67) | | 0.96  (0.25-3.62) | | 1.23  (0.45-3.37) | | Ref. | | 0.25  (0.03-1.97) | | 0.60  (0.21-1.72) | | 0.61  (0.26-1.44) | | Ref. | | 2.19  (0.84-5.69) | | 2.16  (0.85-5.51) | | 1.97  (0.87-4.46) |
| **Be physically active^8^** | |  | |  | |  | |  | |  | |  | |  | |  | |  | |  | |  |
| N | 177 | 109 | | 211 | | 166 | | 126 | | 59 | | 113 | | 110 | | 142 | | 86 | | 184 | | 158 |
| Person-years | 103.7 | 62.3 | | 91.6 | | 87.9 | | 72.5 | | 32.8 | | 54.2 | | 52.4 | | 72.0 | | 48.4 | | 91.3 | | 75.3 |
| No. of deaths | 13 | 7 | | 7 | | 4 | | 18 | | 5 | | 6 | | 1 | | 23 | | 11 | | 12 | | 9 |
| HR  (95% CI) | Ref. | 0.84  (0.33-2.12) | | 0.63  (0.25-1.57) | | 0.35  (0.11-1.08) | | Ref. | | 0.62  (0.23-1.68) | | 0.43  (0.17-1.09) | | **0.08**  **(0.01-0.56)** | | Ref. | | 0.74  (0.36-1.52) | | **0.42**  **(0.21-0.85)** | | **0.39**  **(0.18-0.83)** |
| aHR  (95% CI) | Ref. | 0.77  (0.29-2.01) | | 0.67  (0.26-1.70) | | 0.46  (0.14-1.54) | | Ref. | | 0.87  (0.30-2.47) | | 0.53  (0.20-1.38) | | **0.09**  **(0.01-0.69)** | | Ref. | | 1.05  (0.49-2.28) | | 0.58  (0.28-1.22) | | 0.67  (0.29-1.55) |
| **Limit alcohol consumption^9^** | | |  | |  | |  | |  | |  | |  | |  | |  | |  | |  | |
| N | 45 | 54 | | 177 | | 387 | | 25 | | 23 | | 132 | | 228 | | 30 | | 28 | | 207 | | 305 |
| Person-years | 17.5 | 18.9 | | 102.3 | | 206.8 | | 11.9 | | 8.9 | | 70.1 | | 120.9 | | 8.4 | | 7.9 | | 112.7 | | 157.9 |
| No. of deaths | 2 | 0 | | 7 | | 22 | | 4 | | 1 | | 8 | | 17 | | 0 | | 2 | | 17 | | 36 |
| HR  (95% CI) | Ref. | NE | | 0.56  (0.12-2.73) | | 0.88  (0.20-3.77) | | Ref. | | 0.32  (0.04-2.90) | | 0.34  (0.10-1.12) | | 0.42  (0.14-1.25) | | Ref. | | NE | | NE | | NE |
| aHR  (95% CI) | Ref. | NE | | 0.42  (0.08-2.17) | | 0.67  (0.14-3.12) | | Ref. | | 0.36  (0.04-3.73) | | 0.35  (0.10-1.23) | | 0.35  (0.10-1.19) | | Ref. | | NE | | NE | | NE |
| **Quit smoking^10^** | |  | |  | |  | |  | |  | |  | |  | |  | |  | |  | |  |
| N | 30 | 47 | | 158 | | 428 | | 18 | | 26 | | 107 | | 257 | | 30 | | 35 | | 140 | | 365 |
| Person-years | 20.1 | 23.8 | | 67.9 | | 233.6 | | 11.1 | | 13.3 | | 48.0 | | 139.4 | | 16.7 | | 18.2 | | 66.1 | | 186.0 |
| No. of deaths | 5 | 4 | | 6 | | 16 | | 4 | | 2 | | 6 | | 18 | | 5 | | 2 | | 15 | | 33 |
| HR  (95% CI) | Ref. | 0.67  (0.18-2.49) | | 0.36  (0.11-1.18) | | 0.27  (0.10-0.74) | | Ref. | | 0.41  (0.08-2.22) | | 0.32  (0.09-1.15) | | 0.35  (0.12-1.04) | | Ref. | | 0.37  (0.07-1.88) | | 0.75  (0.27-2.06) | | 0.58  (0.23-1.49) |
| aHR  (95% CI) | Ref. | 0.76  (0.19-3.05) | | 0.41  (0.12-1.47) | | 0.51  (0.16-1.60) | | Ref. | | 0.42  (0.07-2.46) | | 0.37  (0.10-1.36) | | 0.43  (0.13-1.40) | | Ref. | | 0.26  (0.05-1.36) | | 0.84  (0.30-2.35) | | 0.50  (0.18-1.) |
| Abbreviation: N/No., the number of corresponding cases; Ref., reference; aHR, adjusted Hazard Ratios; CI, Confidence Interval; NE, not estimated; BMI, Body Mass Index.  1 The models were adjusted for age, sex (men or women), year post-diagnosis, insurance type (local insured, employee insured or medical-aid beneficiary), income level (decile to tertile; tertile 1 (0,1,2,3,4), tertile 2 (5,6,7), tertile 3 (8,9,10) or missing), and residence (Seoul, metropolitan cities or others).  2 “Unchanged (poor)” refers to individuals whose health behaviors remained poor from pre- to post-assessment (i.e., poor–poor).  3 “Worsen” refers to a worsen in health behaviors, including transitions from moderate to poor, good to poor, or good to moderate.  4 “Enhanced” refers to an enhancement in health behaviors, including transitions from poor to moderate, poor to good, or moderate to good.  5 “Stable (good)” refers to individuals whose health behaviors remained moderate or good from pre- to post-assessment (i.e., moderate-moderate or good-good).  6 Colon cancer was defined as ICD-O-3 codes C18.0 and C18.2-C18.9. Rectum cancer was defined as ICD-O-3 codes C19.9 and C20.9. Cases of multiple primary cancers (n=7) were excluded from the analysis.  7 Participants categorized based on BMI (kg/m²) and waist circumference (cm) - Poor: BMI <19.0 or BMI ≥23.0 with waist circumference ≥90 (men) or ≥80 (women); Moderate: BMI <19.0 or BMI ≥23.0 with waist circumference <90 (men) or <80 (women); Good: BMI between 19.0 and 23.0 with waist circumference <90 (men) or <80 (women).  8 Participants categorized based on the number of days of moderate to vigorous physical activity per week - Poor: 0 days; Moderate: 1-4 days; Good group: ≥5 days.  9 Participants categorized based on the total alcohol consumption - Poor: heavy drinkers (men: ≥2 drink/day, women: ≥1 drink/day); Moderate: light drinkers (men: <2 drink/day, women: <1 drink/day); Good: non-drinkers.  10 Participants categorized based on the smoking status - Poor: current smokers; Moderate: former smokers; Good: non-smokers. | | | | | | | | | | | | | | | | | | | | | | |

## *Supplement Table 5. Sensitivity analysis for the association between behavior changes and the risk of death, followed up from the colorectal cancer diagnosis*

|  | **Stage I (n=1,716)** | | | | | | | **Stage II (n=1,246)** | | | | | | | | **Stage III (n=1,333)** | | | | | | |
| --- | --- | --- | --- | --- | --- | --- | --- | --- | --- | --- | --- | --- | --- | --- | --- | --- | --- | --- | --- | --- | --- | --- |
| **Behavior Changes** | **Unchanged (poor)^2^** | **Worsen^3^** | | **Enhanced^4^** | | **Stable (good)^5^** | | **Unchanged (poor)^2^** | | **Worsen^3^** | | **Enhanced^4^** | | **Stable (good)^5^** | | **Unchanged (poor)^2^** | | **Worsen^3^** | | **Enhanced^4^** | | **Stable (good)^5^** |
| **Be a healthy weight^6^** | |  | |  | |  | |  | |  | |  | |  | |  | |  | |  | |  |
| N | 468 | 292 | | 264 | | 692 | | 350 | | 191 | | 200 | | 505 | | 334 | | 240 | | 236 | | 523 |
| Person-years | 573.4 | 339.0 | | 310.1 | | 826.9 | | 416.6 | | 232.8 | | 240.1 | | 599.5 | | 402.2 | | 280.8 | | 264.7 | | 605.7 |
| No. of deaths | 12 | 9 | | 8 | | 26 | | 25 | | 5 | | 17 | | 34 | | 27 | | 15 | | 28 | | 54 |
| HR  (95% CI) | Ref. | 1.33  (0.56-3.16) | | 1.29  (0.53-3.14) | | 1.55  (0.78-3.07) | | Ref. | | **0.35**  **(0.14-0.92)** | | 1.18  (0.64-2.19) | | **0.95**  **(0.57-1.60)** | | Ref. | | 0.81  (0.43-1.52) | | 1.62  (0.96-2.75) | | 1.35  (0.85-2.15) |
| aHR  (95% CI) | Ref. | 1.42  (0.60-3.39) | | 1.50  (0.61-3.72) | | 1.81  (0.90-3.61) | | Ref. | | 0.43  (0.16-1.12) | | 1.18  (0.63-2.21) | | 1.08  (0.64-1.82) | | Ref. | | 1.00  (0.53-1.89) | | **1.74**  **(1.02-2.96)** | | **1.63**  **(1.02-2.60)** |
| **Be physically active^7^** | |  | |  | |  | |  | |  | |  | |  | |  | |  | |  | |  |
| N | 457 | 288 | | 487 | | 484 | | 374 | | 205 | | 339 | | 328 | | 332 | | 222 | | 408 | | 371 |
| Person-years | 569.7 | 362.7 | | 564.8 | | 552.3 | | 453.6 | | 252.7 | | 406.8 | | 376.0 | | 391.7 | | 262.2 | | 477.5 | | 422.0 |
| No. of deaths | 26 | 13 | | 11 | | 5 | | 38 | | 14 | | 19 | | 10 | | 49 | | 32 | | 23 | | 20 |
| HR  (95% CI) | Ref. | 0.78  (0.40-1.52) | | **0.46**  **(0.23-0.92)** | | **0.21**  **(0.08-0.55)** | | Ref. | | 0.67  (0.36-1.24) | | 0.57  (0.33-1.00) | | **0.33**  **(0.16-0.66)** | | Ref. | | 0.98  (0.63-1.53) | | **0.39**  **(0.24-0.64)** | | **0.38**  **(0.23-0.65)** |
| aHR  (95% CI) | Ref. | 0.86  (0.44-1.68) | | 0.51  (0.25-1.04) | | **0.31**  **(0.12-0.83)** | | Ref. | | 0.79  (0.43-1.47) | | 0.75  (0.43-1.32) | | 0.50  (0.24-1.04) | | Ref. | | 1.23  (0.78-1.95) | | **0.48**  **(0.29-0.79)** | | 0.61  (0.35-1.04) |
| **Limit alcohol consumption^8^** | | |  | |  | |  | |  | |  | |  | |  | |  | |  | |  | |
| N | 140 | 155 | | 421 | | 1,000 | | 75 | | 78 | | 345 | | 748 | | 56 | | 74 | | 440 | | 763 |
| Person-years | 158.4 | 169.8 | | 521.3 | | 1,199.9 | | 91.1 | | 98.8 | | 411.6 | | 887.6 | | 57.8 | | 78.7 | | 526.8 | | 890.2 |
| No. of deaths | 3 | 3 | | 13 | | 36 | | 6 | | 3 | | 23 | | 49 | | 1 | | 4 | | 40 | | 79 |
| HR  (95% CI) | Ref. | 0.95  (0.19-4.70) | | 1.26  (0.36-4.43) | | 1.54  (0.47-4.99) | | Ref. | | 0.45  (0.11-1.80) | | 0.85  (0.35-2.10) | | 0.83  (0.36-1.95) | | Ref. | | 2.89  (0.32-25.89) | | 4.09  (0.56-29.74) | | 4.84  (0.67-34.77) |
| aHR  (95% CI) | Ref. | 0.70  (0.14-3.48) | | 0.94  (0.27-3.33) | | 0.87  (0.26-2.88) | | Ref. | | 0.54  (0.13-2.18) | | 0.84  (0.34-2.09) | | 0.61  (0.26-1.44) | | Ref. | | 2.30  (0.32-16.80) | | 2.10  (0.23-19.01) | | 2.30  (0.32-16.80) |
| **Quit smoking^9^** | |  | |  | |  | |  | |  | |  | |  | |  | |  | |  | |  |
| N | 80 | 109 | | 394 | | 1,133 | | 47 | | 57 | | 282 | | 860 | | 57 | | 73 | | 305 | | 898 |
| Person-years | 104.8 | 129.4 | | 471.4 | | 1,343.8 | | 57.3 | | 68.1 | | 328.3 | | 1,035.3 | | 67.6 | | 91.8 | | 343.6 | | 1,050.4 |
| No. of deaths | 5 | 6 | | 12 | | 32 | | 10 | | 3 | | 15 | | 53 | | 9 | | 4 | | 32 | | 79 |
| HR  (95% CI) | Ref. | 1.01  (0.31-3.32) | | 0.58  (0.20-1.64) | | 0.54  (0.21-1.38) | | Ref. | | **0.26**  **(0.07-0.96)** | | **0.28**  **(0.13-0.62)** | | **0.30**  **(0.15-0.59)** | | Ref. | | 0.32  (0.10-1.04) | | 0.73  (0.35-1.53) | | 0.57  (0.29-1.14) |
| aHR  (95% CI) | Ref. | 0.86  (0.26-2.88) | | 0.62  (0.21-1.78) | | 0.48  (0.18-1.25) | | Ref. | | **0.17**  **(0.05-0.65)** | | **0.22**  **(0.10-0.50)** | | **0.19**  **(0.09-0.39)** | | Ref. | | **0.23**  **(0.07-0.76)** | | 0.80  (0.38-1.67) | | **0.43**  **(0.21-0.86)** |
| Abbreviation: N/No., the number of corresponding cases; Ref., reference; aHR, adjusted Hazard Ratios; CI, Confidence Interval; BMI, Body Mass Index.  1 The models were adjusted for age, sex (men or women), year post-diagnosis, insurance type (local insured, employee insured or medical-aid beneficiary), income level (decile to tertile; tertile 1 (0,1,2,3,4), tertile 2 (5,6,7), tertile 3 (8,9,10) or missing), and residence (Seoul, metropolitan cities or others).  2 “Unchanged (poor)” refers to individuals whose health behaviors remained poor from pre- to post-assessment (i.e., poor–poor).  3 “Worsen” refers to a worsen in health behaviors, including transitions from moderate to poor, good to poor, or good to moderate.  4 “Enhanced” refers to an enhancement in health behaviors, including transitions from poor to moderate, poor to good, or moderate to good.  5 “Stable (good)” refers to individuals whose health behaviors remained moderate or good from pre- to post-assessment (i.e., moderate-moderate or good-good).  6 Participants categorized based on BMI (kg/m²) and waist circumference (cm) - Poor: BMI <19.0 or BMI ≥23.0 with waist circumference ≥90 (men) or ≥80 (women); Moderate: BMI <19.0 or BMI ≥23.0 with waist circumference <90 (men) or <80 (women); Good: BMI between 19.0 and 23.0 with waist circumference <90 (men) or <80 (women).  7 Participants categorized based on the number of days of moderate to vigorous physical activity per week - Poor: 0 days; Moderate: 1-4 days; Good group: ≥5 days.  8 Participants categorized based on the total alcohol consumption - Poor: heavy drinkers (men: ≥2 drink/day, women: ≥1 drink/day); Moderate: light drinkers (men: <2 drink/day, women: <1 drink/day); Good: non-drinkers.  9 Participants categorized based on the smoking status - Poor: current smokers; Moderate: former smokers; Good: non-smokers. | | | | | | | | | | | | | | | | | | | | | | |

Supplement Figure 1. Hazard ratios for the risk of death from postdiagnosis BMI among colorectal cancer survivor
